# Tracing the botanical origins of UK heather honey by relative quantification of plant DNA

**DOI:** 10.1038/s41538-025-00561-1

**Published:** 2025-09-30

**Authors:** Sophie Dodd, Zoltan Kevei, Zahra Karimi, Jane Jennifer Suresh Kumar, Anastasios Koidis, Maria Anastasiadi

**Affiliations:** 1 Centre for Soil, Agrifood and Biosciences, Faculty of Engineering and Applied Sciences, Cranfield University, Cranfield, UK; 2 Institute for Global Food Security, School of Biological Sciences, Queen’s University of Belfast, Belfast, UK

**Keywords:** Molecular biology, Plant sciences

## Abstract

Heather honey is an important honey type produced in the UK, valued for its unique flavour, thixotropic texture and health-promoting properties. Botanical authentication can be challenging due to the natural variability in honey composition and typical pollen analysis relies heavily on expert knowledge. As an alternative, real-time PCR (qPCR) can be a rapid and robust method to identify floral species in honey. In this work, species-specific markers for *Calluna vulgaris* and *Erica cinerea* were developed and used to quantify 266 honey samples relative to the plant *trnL* P6 loop. The method classed 96% of 234 heather honeys as containing > 3% heather DNA, with 68% classified as dominant (> 45%) for ling heather origin. Moreover, high specificity was achieved with negligible amplification in the 32 non-heather honeys. Our qPCR method offered comparable results to melissopalynology, DNA metabarcoding, and digital PCR, showing potential as an alternative and accessible method for botanical authentication of heather honeys.

## Introduction

Heather honey can describe honey that is derived from plants in the *Ericaceae* family^1^. Heather typically grows in acidic soil but can flourish on a broad range of climates, altitudes and soil types, and is widespread in many countries particularly in Northwest Europe^2^. In the UK the dominant heather species are ling heather (*Calluna vulgaris*) and bell heather (*Erica cinerea*), which can typically be found at heathlands and moorlands across the country and contribute to one third of total nectar production in the UK^3^. In fact, *C. vulgaris* is a vital forage source in the late summer, when there are fewer flowering plants available^4^. As ling heather produces a high volume of nectar, it is common practice for bee farmers to move hives close to the heather moors at the end of summer to provide sufficient forage for the bees and produce principally heather honey, making it one of the most important and valuable honey types produced in the UK^5^. Heather honey is highly nutritious and appreciated for its unique flavour, texture and appearance^6^. *C. vulgaris* in particular is valued as a source of bioactive components, with heather honeys exhibiting a wide range of biological activities^7^, contributing to its popularity and higher market value. However, ling heather honeys are complicated to extract due to their thixotropic nature, meaning they are more time-consuming and expensive to produce than other honeys^8^. Therefore, it is imperative to provide accessible and cost-effective ways to authenticate heather honey, to prove the product value and maintain the economy of the honey market.

Despite possessing distinct properties, authenticating heather honey based on physicochemical parameters alone can be misleading, as honey is rarely derived from a single flora. The presence of different nectar sources directly affects the honey composition, creating variation in standard measurements which are common across different honey types^4^. Furthermore, the extraction technique, postharvest processing and storage conditions can additionally affect the physicochemical appearance^9,10^. The established way to identify the botanical sources of honey is based on pollen analysis using light microscopy, known as melissopalynology^11^. Pollen in honey is a by-product of nectar foraging, or collected by the bees to fulfil nutritional requirements, thus it can offer a representative fingerprint of the honeys origins^12,13^. However, this requires a highly specialised method involving the counting and identification of individual pollen grains and relies heavily on experienced analysts with the ability to provide correct identification and interpretation of the results^14^.

DNA analysis has been proposed as an alternative way to detect plant species in honey, and DNA markers have been developed for many different plant species to authenticate their botanical origins using real-time PCR (qPCR)^15–17^. The application of high-resolution melting (HRM) curves was applied to help differentiate between genetically similar species, such as for lavender honey^18^, or used to identify popular honey types with a common marker (*rbcL*)^19^. However, the interpretation of HRM in samples with mixed species may be problematic and the quantification of DNA sources in honey using qPCR methodologies has been rarely attempted. Advanced metabarcoding methods using DNA sequencing have been employed to identify the entirety of plant species in honey samples^20–23^, but these methods are resource-intensive and require complex bioinformatic analysis, meaning they may not be widely accessible for the botanical authentication of honey.

Relative quantification has been associated with qPCR methods and used to quantify proportions of animal species in meat, fish and dairy products with different universal markers targeting nuclear or mitochondrial gene regions^24–30^. In addition, the methods have been employed to identify different plant-based products, such as for protein powder^31^, herbal supplements^32^, bakery products^33^ and spices^34^ to authenticate or detect allergens. However, qPCR methods utilising relative quantification have not yet been applied to honey and have the advantage of circumventing standard curves. This is of particular value when dealing with food products where appropriate reference material is not accessible or comparable due to unknown factors such as raw material processing^35^.

The aims of this study were to design DNA markers to distinguish between *C. vulgaris* and *Erica* species of heather and attempt a relative quantification by employing the conserved *trnL* P6 marker as a plant DNA reference. The accuracy of the method was assessed by comparing traditional and DNA-based methods for pollen analysis. Finally, a conclusive honey classification was provided to characterise the floral source of heather honeys from across the UK.

## Results

### DNA marker specificity

Initially, a comprehensive in silico analysis was performed to assess marker specificity, revealing that no other species were an exact match to either of the primer pairs (Supplementary Data 2 and 3). The developed specific makers were tested on *C. vulgaris* and *E. cinerea* plant extracts from different regions of the UK using qPCR, to ensure sufficient amplification success and specificity (Table S2).

All *C. vulgaris* and *E. cinerea* extracts amplified the respective target marker, with samples from leaf cuttings generally exhibiting lower amplification (higher C_q_ value). The highest C_q_ value obtained for the respective target marker was C_q_ 22.51 for sample CV11 from the South West. Amplification for the non-target marker was seen in five out of twelve *C. vulgaris* samples and five out of eight *E. cinerea* samples ranging from C_q_ 33.75–37.13 for the EC_trnL marker in *C. vulgaris* extracts and C_q_ 25.46–36.55 for the CV_trnL marker in *E. cinerea* samples.

Marker amplification in other non-target plants was minimal (Table S3) and was evaluated after relative quantification.

### Relative quantification of heather

**Fig. 1:**
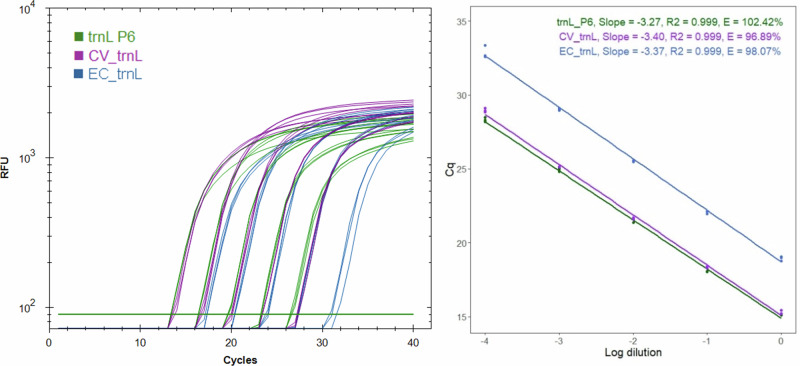
Primer efficiencies. Amplification of trnL P6 , CV_trnL , and EC_trnL  markers with a standard curve of honey extract (1X – 10,000X diluted). The raw data (RFU) and regression (C_q_) are marked. The three technical replicates are shown for each reaction.

The primer efficiencies were calculated using a standard curve of Scottish ling honey sample (H-S-84), which showed a low C_q_ value for all markers (Fig. 1). The markers respectively had efficiencies of 102.42%, 96.89% and 98.07% for trnL_P6, CV_trnL and EC_trnL. Furthermore, all markers showed good linearity with R^2^ values of 0.99.

The${\,2}^{-\Delta {Ct}}$ relative quantity (RQ) was calculated for the different types of plants obtained in this study with qPCR using Eq. (2), to test for marker specificity on flora which could be expected to be found in heather honey (Table 1). The obtained C_q_ values for all markers used for the calculation can be found in Table S3.

**Table 1:** Relative quantity (RQ) of *Calluna vulgaris* (CV_trnL) and *Erica cinerea* (EC_trnL) DNA marker in plant extracts, relative to the plant *trnL* P6 loop

ID	species	CV_trnL		EC_trnL	
		RQ	SD	RQ	SD
CV05	*Calluna vulgaris*	**1.20**	0.04	0.00^b^	0.00^b^
EC01	*Erica cinerea*	0.00^b^	0.00^b^	**1.19**	0.10
RW	*Chamaenerion angustifolium*	0.00^b^	0.00^b^	0.00^b^	0.00^b^
Th	*Cirsium* or *Carduus* spp.	0.00^b^	0.00^b^	0.00^b^	0.00^b^
ET	*Erica tetralix*	^a^	^a^	0.66	0.04
Mi	*Mentha spp*.	0.00^b^	0.00^b^	0.00^b^	0.00^b^
PE	*Potentilla erecta*	^a^	^a^	^a^	^a^
PV	*Prunella vulgaris*	^a^	^a^	^a^	^a^
RF	*Rubus fruticosus*	0.00^b^	0.00^b^	0.00^b^	0.00^b^
SC	*Silene coronaria*	0.00^b^	0.00^b^	0.00^b^	0.00^b^
SP	*Succisa pratensis*	0.00^b^	0.00^b^	0.00^b^	0.00^b^
Da	*Taraxacum spp*.	0.00^b^	0.00^b^	0.00^b^	0.00^b^

The markers were amplified using qPCR with three technical replicates, where a RQ of 1 indicates equal amplification.

^a^not amplified ^b^RQ < 0.001.

For the target plants the C_q_ values of the reference plant marker and the target marker were very similar; for *C. vulgaris* the C_q_ values were 20.41 and 20.15, and for *E. cinerea* they were 21.16 and 20.90 respectively **(**Table S3). In both cases the target plant marker was amplified in slightly fewer cycles than the reference plant marker, resulting in RQ values of 1.20 ± 0.04 for *C. vulgaris* and 1.19 ± 0.10 for *E. cinerea*. Although in both cases the non-target marker was amplified (C_q_ 37.63 for *C. vulgaris* and C_q_ 31.90 for *E. cinerea*), the amplification of the general plant marker was much stronger, resulting in an RQ of < 0.001 for the non-target marker for both plants. Interestingly, the *E. tetralix* sample amplified the *E. cinerea* marker significantly, resulting in an RQ of 0.66 ± 0.04, suggesting that the bell heather marker may also amplify closely related *Erica* species. None of the other plant extracts amplified the CV_trnL or EC_trnL marker significantly, with RQ values of < 0.001 when amplification was seen.

To take into account the slightly increased amplification of the target markers compared to the reference plant marker seen in the target plant controls (Tables 1, S3), the corrected RQ $({2}^{-\triangle \triangle {Ct}})$ was calculated for each honey sample by applying the plant ${2}^{-\triangle {Ct}}$ values as control calibrators to the respective ${2}^{-\triangle {Ct}}$ RQ in the honey extract (Table S4) as described in Eq. (3).

The mean RQ of *C. vulgaris* in the 234 heather honeys was 0.57 ± 0.31, and the mean RQ of *Erica* spp. was 0.03 ± 0.07 indicating that the majority of heather honey samples contained ling heather as a major floral source rather than *Erica* spp. The two samples from the Republic of Ireland (ROI) did not amplify either heather marker above 0.03 RQ, suggesting that neither ling nor bell heather was the major floral source. For the 32 non-heather honeys the mean RQ of *C. vulgaris* was 0.00 ± 0.00, and the mean RQ of *Erica* spp. was 0.00 ± 0.01, indicating that none of these honeys contained either ling or *Erica* spp. heather.

### Pollen analysis by melissopalynology

To confirm the results of the developed DNA marker test, four reference samples were sent for traditional pollen analysis via microscopy. *C. vulgaris* was identified in all four of the honey samples sent for melissopalynology at 7%, 25%, 1% and < 1% respectively for H-S-05, H-Y-05, H-SW-02 and H-SW-03. *E. cinerea* pollen grains were not specifically identified, so pollen originating from bell heather would fall under the family group Ericaceae (excluding *C. vulgaris* as this was a separate species category). Pollen from the Ericaceae group was detected in the two samples classified as bell heather, containing honey but with low levels of < 1% and 2% for H-SW-02 and HS-SW-03, and not detected at all in H-S-05 and H-Y-05.

Pollen coefficients (PCs) were applied to the melissopalynology results for each pollen type with ≥ 1% abundance. For *C. vulgaris* and Ericaceae, the PC values were 12 and 10, where the PC for a normally represented flora was 50. After correction, the abundance of *C. vulgaris* in honey samples H-S-05, H-Y-05 and H-SW-02 increased to 31%, 60% and 4%, and for honey sample H-SW-03, the presence of Ericaceae rose to 9%.

The full results from the melissopalynology analysis can be found in Supplementary Data 4.

### Pollen analysis by metabarcoding

The same four samples underwent amplicon sequencing of the *trnL* P6 region to compare with the melissopalynology and PCR analysis. The number of reads matched to *C. vulgaris* and *Erica* spp. was divided by the total number of reads for each honey sample to calculate the species or genus relative abundance. In samples H-SW-02 and H-SW-03 *E. tetralix* was detected at 2 and 3 reads, which were grouped with the *E. cinerea* reads. No other *Erica* sp. was detected in the samples.

*C. vulgaris* DNA was detected in all four of the honey samples at 23% (H-S-05), 24% (H-Y-05), < 1% (H-SW-02) and < 1% (H-SW-03). Similar to melissopalynology, *Erica* spp. were detected at 1% and 2% in H-SW-02 and H-SW-03 and not detected at all in H-S-05 and H-Y-05.

Due to the comparable abundances of pollen detected in melissopalynology and DNA metabarcoding, the pollen coefficient values were applied to the metabarcoding results. This was done by matching all reads to an appropriate taxon and grouping according to the categories used for melissopalynology. If the DNA identified taxa over 1% abundance which was not present in pollen analysis, then a new category was included for that plant family. The correction analysis was only done on samples presenting over 1% of reads. After correction, the abundance of *C. vulgaris* increased to 47% and 58% for H-S-05 and H-Y-05, and for honeys H-SW-02 and H-SW-03, the presence of *Erica* spp rose to 7% and 9% respectively.

The full results from metabarcoding analysis can be found in Supplementary Data 4.

### Comparison of methodologies

**Fig. 2:**
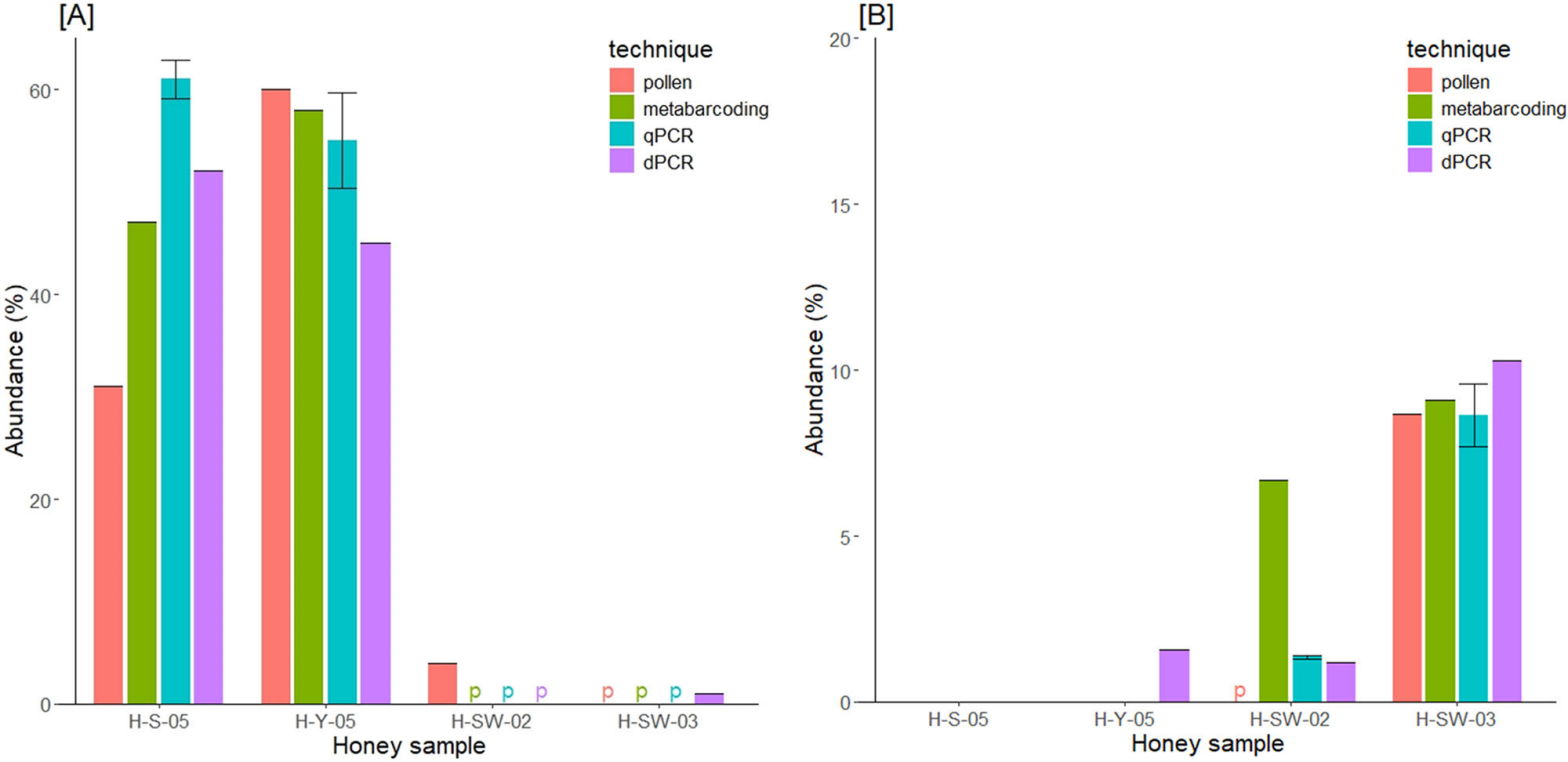
Results from melissopalynology, metabarcoding, qPCR and dPCR for detection of *C. vulgaris* and *Erica* spp. Comparison of methods used to quantify [A] *Calluna vulgaris* and [B] *Erica* spp. in four UK heather honey samples where “p” indicates a presence at < 1%. The methods used were: pollen (melissopalynology) (), metabarcoding (), qPCR () and dPCR (). Data for pollen and metabarcoding were corrected using pollen coefficent values. Error bars for qPCR are shown from three technical replicates. No replicates were included for the other techniques.

The corrected melissopalynology and metabarcoding results were used to determine the accuracy of the qPCR method and compared with results from dPCR (Fig. 2, Table S5). Results from qPCR and dPCR with an RQ under 0.001 (0.1%) were classed as not detected.

The results for all methods were highly comparable, with mean values of 48% ± 13% and 54% ± 7%, for quantification of *C. vulgaris* in H-S-05 and H-Y-05 and mean values of 3% ± 3% and 9% ± 1% for quantification of *Erica* spp. for H-SW-02 and H-SW-03, respectively.

### Honey classification

**Fig. 3:**
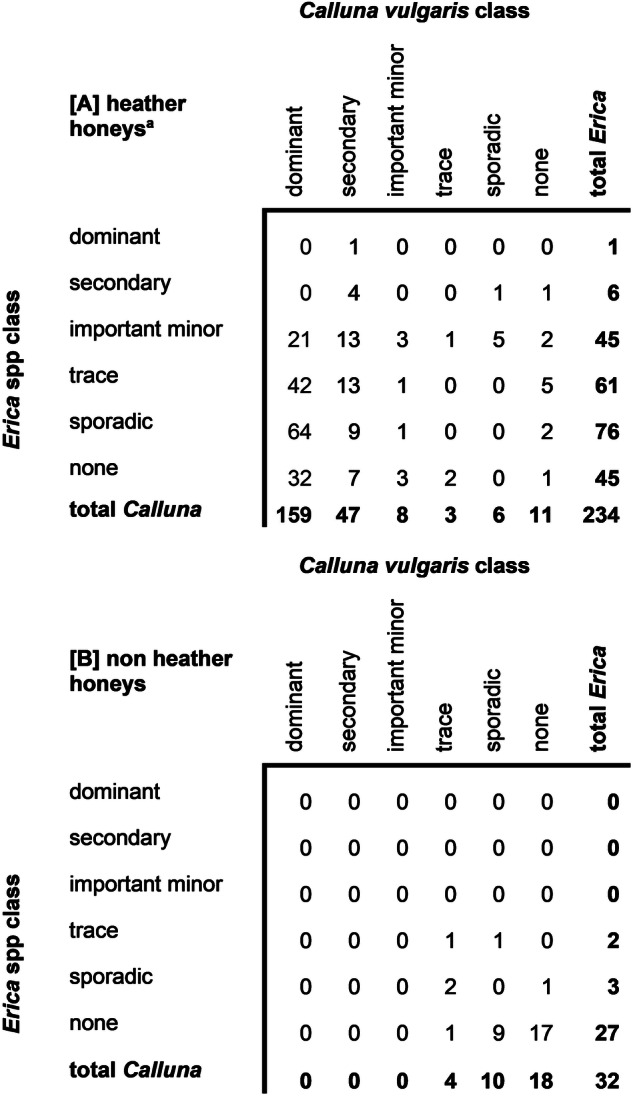
Classification of heather and non-heather honey. Classification of honey based on relative quantity (RQ) of *C. vulgaris* and *Erica* spp. pollen in [A] heather and [B] non-heather honey types. The number of honeys belonging to each class is displayed for *C. vulgaris* (columns) and *Erica* spp. (rows) where class is defined as by RQ values; dominant: > 0.45, secondary: 0.16–0.45, important minor: 0.03–0.16, trace: 0.03–0.01, sporadic: 0.01–0.001 and none: < 0.001. ^a^heather honeys are honeys with a suspected source of heather flowers nearby the apiaries.

Each honey sample was classified according to the abundance of *C. vulgaris* and *Erica* spp. DNA calculated using the qPCR method for relative quantification as following: dominant: > 0.45, secondary: 0.16–0.45, important minor: 0.03–0.16, trace: 0.03–0.01, sporadic: 0.01–0.001 and none: < 0.001 (Fig. 3).

According to this classification criteria 68% of the heather honey samples showed a dominant source of heather (*C. vulgaris:* 159, *Erica* spp: 1) and could be confidently classified as monofloral heather honeys. A further 28% of the samples were classed as having secondary (21%) or important minor (7%) pollen sources from heather, totalling 96% of the heather honey sample set. The remaining 4% (*n* = 10) heather honeys were classified as ‘trace’, ‘sporadic’ or ‘none’ for either *C. vulgaris* or *Erica* spp pollen, with only one sample classed as containing no heather DNA at all.

Considering the non-heather honey samples, none of them were classified as containing more than trace amounts of heather DNA, with the highest RQ values observed as 0.015 for both *C. vulgaris* and *Erica* spp. in separate samples.

## Discussion

To obtain accurate plant specificity, the chloroplastid *trnL* gene region was selected as a candidate for marker design as it is known for highly conserved regions (typically coding regions), but also hypervariations, such as intergenic spacers and introns^36^. The *trnL* p6 loop was selected as a reference marker as the oligos sites are highly conserved in Angiosperms and Gymnosperms, and produce a short PCR product (< 100 bp) which is suited to test typically degraded DNA when extracting from processed food products such as honey^37^. Moreover, the *trnL* P6 region has already shown good amplification success in honey samples using both qPCR and metabarcoding approaches^20,38^.

Other genes such as ITS, *matk* and *rbcL* were considered as key regions for marker design, but it was highly important to use a target and reference marker designed on the same gene region to account for possible variabilities in the copy number of plastid and ribosomal DNA content in pollen of different species^39,40^. Therefore, designing specific DNA markers on the *trnL* region would take into account the likelihood of variable copy numbers in samples of mixed species, assuming that the reference (*trnL* P6) and target *trnL* regions were present in equal copies in the plant (Fig. S1).

Specific DNA markers were designed on hypervariable regions to differentiate between ling heather (*Calluna vulgaris)* and bell heather (*Erica cinerea*), and the target markers were amplified in ling and bell heather plant DNA extracts taken from different regions of the UK, including those from apiary sites (Table S2). Thus, we demonstrated the suitability of the markers to amplify in a variety of heather plants taken from around the UK and honey samples taken from regions across the countries. However, amplifications were observed for the specific markers in non-target plants (Tables 1, S2, S3), for example, the *C. vulgaris* specific marker amplified in some *E. cinerea* samples, with the lowest C_q_ value of 25.46. Nonetheless, the amplification of the *E. cinerea* target marker in the same extracts was amplified in > 11 cycles fewer, indicating that the non-target amplification was negligible and would not appear as false positive result once the relative quantification analysis was completed, as shown in Table 1. In fact, the highest non-target RQ value was 0.0006 for EC01, therefore it was assumed that any amplification < 0.001 was non-specific for the presence of *C. vulgaris* or *Erica* spp. DNA in the sample.

Likewise, the RQ values of other non-target plants (Table 3), except *E. tetralix*, taken from the heather apiary sites were > 0.001 (Table 1), indicating that other flora is not amplified in the heather honeys by the target markers, although this was by no means a complete assessment of all possible forage sources. Indeed, the in-silico primer-BLAST analysis revealed 501 species that had between 3 and 6 mismatches to the CV_trnL marker, and 432 species that had between 1 and 6 mismatches to the EC_trnL primer pair, suggesting that there was a chance of non-target binding with these species (Supplementary Data 2 and 3).

Hence, the suitability of the markers was further tested by inclusion of 32 non-heather honeys containing a rich diversity of UK flora (Table S3), to assess whether cross-reactivity would be seen in typical UK honey forage sources. In these samples, minimal non-target amplification was seen with a maximum RQ of 0.015 for both the CV_trnL and EC_trnL markers, confirming the suitability of the markers for UK heather honey identification (Table S4). However, if used on non-UK honeys, it would be strongly suggested that the markers should be evaluated for specificity on a broader range of non-target plants, particularly where potential floral sources have not been included in this study.

Notably, the *E. cinerea* target marker amplified strongly (C_q_ 17.55) in the related *E. tetralix* sample, which flowers at a similar time to bell and ling heather whilst appearing in comparable locations^3^. However, it is not expected to have the cross-leaved heath in honey samples due to its flower shape affecting the honeybee’s ability for nectar collecting^41^. The in-silico BLAST search revealed that the *E. cinerea* marker (EC_trnL) showed only one sequence mismatch on the third nucleotide from the 3’ end of the reverse primer compared with 155 *Erica* species, so it is not surprising that the *E. cinerea* marker binds to the *E. tetralix* samples (Fig. S2, Supplementary Data 3). In this case, the *E. cinerea* marker could be used as a marker for *Erica* spp., rather than being specific to *E. cinerea*, although further testing should be done to fully establish which plants could be detected.

Despite this, the specific *trnL* markers offered the separation of *C. vulgaris* from *Erica* species, which was not accomplished by previously proposed DNA markers for heather honey authentication^15^. Whilst the marker specificity could be improved by using TaqMan probes, this approach would not be suitable for the reference *trnL* P6 marker. For this reason, specific probes were not explored in this study, as it was not realistic to compare amplification from different qPCR approaches (SYBR Green and TaqMan).

Several studies successfully used genomic or chloroplast DNA markers to detect specific floral species in food products. For example, incense (*Pittosporum undulatum*) DNA in Portuguese honey samples was quantified down to 0.01 pg by using a standard curve of incense genomic DNA^16^. Another study used the 18S rRNA universal marker as a control to achieve relative quantification of safflower adulteration in saffron^34^. Nevertheless, as the 18S rRNA marker is highly conserved amongst eukaryotes^42^, the marker can amplify DNA from other sources in honey (e.g. bee DNA) and would not provide an accurate representation of the total plant DNA in a sample, hampering the use for relative quantification studies where multiple sources of DNA are expected in the sample.

The ${2}^{-\triangle \triangle {Ct}}$ method was used to calculate the RQ of *C. vulgaris* and *Erica* spp. in the honey samples, which assumes uniform primer efficiency. The markers used in this study produced efficiencies between 96.9–102.4% (Fig. 1) indicating the formula was suitable for use. Despite the similar primer efficiencies, the ${2}^{-\triangle {Ct}}$ RQ of the *C. vulgaris* and *E. cinerea* plant extracts revealed that the target marker was amplified at a slightly lower cycle number than the universal plant marker, with RQ values of 1.20 and 1.19, respectively. Therefore, the amplification of the relevant plant DNA sample was used as the control in the ${2}^{-\triangle \triangle {Ct}}$ RQ equation, to normalise the results and account for the slightly higher RQ observed in reference plant material. Moreover, applying normalisation can help to account for instrument variations by including positive control extracts in every assay, thus, this equation may be preferred to the ${2}^{-\triangle {Ct}}$. Nevertheless, if no appropriate reference material is available, the ${2}^{-\triangle {Ct}}$ for relative quantification can still be applied with comparable accuracy, such as was done for tuna authentication^24^.

However, even after correction, the RQ of *C. vulgaris* DNA in honey occasionally resulted in a value higher than 1 (Table S4). For example, the highest RQ obtained was 1.67 ± 0.31 for sample H-S-11, which had a C_q_ value of 17.74 ± 0.10 and 16.73 ± 0.06 for trnL_P6 and CV_trnL, respectively. In total, RQ > 1.00 was observed in 13 cases (8%) of the 159 honey samples which were assigned as dominant for ling heather; thus there was no prominent issue with the calculation method. In fact, the ${2}^{-\triangle \triangle {Ct}}$ method has been criticised for over-estimating the relative quantification with 100% primer efficiencies^43^, therefore it has been proposed to calculate the individual efficiency for each sample, which could lead to a more accurate relative quantification result^44^. Indeed, in this work, the primer efficiencies were calculated based on only one DNA extraction, and could be affected by the presence of PCR inhibitors such as polyphenols, enzymes, wax particles or organic acids derived from the individual honey sample^45^.

Besides PCR inhibitors, the reliance on the reference marker to exactly match each target species in the sample is imperative and would be affected by the primers’ efficiency. Any mismatches, especially at the 3’ ends of the oligo pairs, could cause increased C_q_ values with decreased amplification efficiency. For example, in a meat quantification study, it was found that the duck sequence had a mismatch on the third nucleotide from the 3’ end of the *myostatin* reference primer, resulting in considerably higher C_q_ values (~8–10 cycles difference) for duck than species, which exactly matched the primer sequence^46^. Although in the case of samples including a mixture of many taxa, such as honeys, the effects of the inclusion of species having mismatches in their primer sequences would probably not be as substantial, as one would expect that the majority aligns with the selected primers. But even one species in the mix with a single nucleotide difference could result in a small increase to the C_q_ value, as we have observed in this study. However, these variations may only increase the RQ by a small margin, which is unlikely to affect the final honey classification and therefore is not of immediate concern when assessing dominant floral sources.

The ability to generate quantitative results from pollen analysis using plastid DNA has been questioned as the variations in the copy number of plastid in pollen are not well understood^47^. Additionally, it is known that DNA analysis of mixed samples can be significantly biased by the amplifications, generating quantification limitations^21^. However, several studies have shown relatively good correlations between pollen microscopy and DNA metabarcoding from both honey^23,48^ and environmental pollen samples^39,49,50^, although this depended on the investigated taxa. Furthermore, it was shown that the *trnL* P6 region analyses presented an excellent correlation between the amplicon DNA reads and pollen grain abundance, indicating that the marker was not subject to PCR bias^51^. To check the accuracy of the implemented qPCR method, we have compared the traditional melissopalynology with the DNA metabarcoding of the *trnL* P6 loop and dPCR results using reference honey samples (Fig. 2).

The *trnL* P6 loop marker is known for covering a wide taxonomic breadth; however, this also means that the taxonomic resolution is lower, rather identifying genus or family level than species^52^. Fortunately, by sequencing the *trnL* P6 loop, we were able to taxonomically distinguish *C. vulgaris* and *E. cinerea*, and also other members of the *Erica* genus or Ericaceae family. Interestingly, metabarcoding identified two reads of *E. tetralix* in H-SW-02 and three reads in H-SW-03, which were grouped together with the *E. cinerea* reads for method comparison purposes. The metabarcoding did not identify any other Ericaceae plants, except in the H-S-05 sample, where *Vaccinium vitis-idaea* was identified at 2.1% abundance, which can be expected at heather moors^53^. However, this was different from the melissopalynology results, where no Ericaceae plants were detected for sample H-S-05 (Supplementary Data 4). Based on the good comparability between the used methods (Fig. 2), we can assume that the positive melissopalynology results for Ericaceae in H-SW-02 and H-SW-03 were derived from *Erica* spp., with the majority from bell heather.

The main discrepancy between the method results was for *C. vulgaris* in H-S-05, where the pollen analysis gave an abundance of 31% compared to 47– 61% with the DNA methods. This could be attributed to the heterogeneous nature of honey, as the replicability of both melissopalynology and metabarcoding can be variable, with 28% similarity for melissopalynology and 64% similarity for metabarcoding results observed for two honey samples taken from the same hive^21^. However, the high variability obtained in that study could be explained by the sampling strategy and differences in methodology, as only 2 g of honey was used for melissopalynology compared to 10 g for DNA analysis. Furthermore, the number of samples compared was low (*n* = 2) and the tested honey contained a much longer list of plants with smaller proportions rather than containing obvious dominant plant sources.

Indeed, in our study, no biological replicates were performed for either discussed techniques, as it is not a typical practice for pollen analysis. As an excellent alternative, the qPCR method offers similar accuracy with fewer resources and the ability to process large sample numbers, but can only provide information on selected species, where suitable target markers are available. The abundance of *C. vulgaris* and *Erica* spp. calculated by the relative quantification of target DNA markers provided very similar values to those expected based on pollen analysis, with a standard deviation of no more than 13%, demonstrating the suitability of the qPCR method as an alternative to the melissopalynological approach.

Digital PCR uses partitioning within the PCR reaction to produce an absolute copy number result based on the fluorescent imaging of positive and negative droplets with Poisson statistics^54^. The method is commended for its ability to provide appropriate quantification without the need for standard curves or reference material and is more tolerant to PCR inhibitors^55^. We found that the RQ of *C. vulgaris* and *Erica* spp were generally lower using dPCR, which could account for the suspected over-estimation of the ${2}^{-\triangle \triangle {Ct}}$ method, as discussed above. However, it is not critical to use the higher resolution dPCR for authentication of dominant floral sources of the honey, as we found minor deviation between the techniques. The dPCR methodology is more suited where increased sensitivity is the main goal, such as for adulterant detection^56^.

Honey classification based on pollen analysis is complex, as pollen can be over- or under-represented in different plants, thus in the honey as well. Ling heather can be underrepresented with pollen ranging from 10 – 77% in the unifloral honey, where *Erica* spp. are suggested to be normally represented at > 45% for monofloral classification^14^. In Croatia and Serbia the minimum purity percentage for ling heather pollen content in honey needs to be 20%^57^, for Poland it is 45%^58^. However, in the UK, no specific guidelines exist for the amount of *C. vulgaris* or *Erica* spp. pollen required to authenticate heather honey. Indeed, in our previous work, we found *C. vulgaris* pollen present in heather honey samples ranging from 3 – 77%, demonstrating the expected variability of ling pollen counts in UK honey samples^59^.

This problem can be overcome by using pollen coefficients (PCs), which were developed to normalise the expected amount of pollen in honey samples by taking into account how much of each pollen types are represented based on the flower pollen content of each species^60^. Based on this, ling heather pollen can be represented around four times less than species that represent pollen normally, such as white clover (*Trifolium repens*), which should be taken into account during melissopalynological analysis^61^. Interestingly, Sawyer notes that *Erica* spp., such as bell heather, are more underrepresented than ling heather, which is in line with beekeepers’ observations (personal communication) while contrary to research data^62^.

In order to apply PCs, information about the abundance of species pollen diversity in the sample is a prerequisite to adjust for both under and overrepresented species. Therefore, PCs were not applied to the qPCR or dPCR methods, as there was no data available on the identification or abundance of other plant species in the sample. According to the method comparison (Fig. 2), the RQ of both techniques was highly comparable to the corrected melissopalynological results without applying the PC, suggesting that correction is not necessary when using a specific target marker.

Since the qPCR results were similar to the corrected pollen analysis, it was deemed suitable to use normal classification metrics typically employed to classify the abundance of pollen in a honey sample. Therefore, if samples with > 45% abundance (RQ > 0.45) of ling classed as monofloral, then 159 out of 234 samples (68%) were monofloral ling heather honeys, and one sample (0.4%) was a monofloral bell heather honey. However, this bell heather honey originating from Wales also contained ling as a secondary component (ling: 0.31, bell: 0.50). In this case, the honey can be classified based on which heather type had a higher abundance.

Alternatively, the abundance from both markers can be added together to present the total heather class. For instance, in H-S-82, the *C. vulgaris* RQ was 0.43, which would not meet the threshold for monofloral ling heather honey; however, if the *Erica* spp. RQ of 0.05 was added, the sample would be over 0.48 abundance of total heather and classified as a monofloral heather honey. If the goal was to detect heather without distinguishing species, then a more general heather marker can be used, such as the one designed for the *adh1* gene region^15^, with the caveat that the relative quantification method developed here may not be suitable for genomic markers with lower copy number present, as we have tested for the plastid *trnL* region.

Our method revealed a much higher prevalence of *C. vulgaris* compared to *Erica* spp in UK heather honey samples, which could be due to the fact that *Erica* species are preferred by bumblebees, whilst honeybees favour *C. vulgaris*^3^. As *Erica* species flowers earlier than *C. vulgaris*, by the time beekeepers have moved hives close to the heather field, the *Erica* flowers are already reduced or foraged by other pollinators. Another contributing factor can be the reported decline of *Erica* spp. as bee forage within the British landscape over the last few decades^63^, which could explain why *Erica* spp. are rarely classed as a dominant or secondary pollen source in the studied heather honeys.

According to our classification, 15 out of the 32 non-heather honey samples contained heather DNA at trace or sporadic amounts ( < 0.03), where the remaining samples had no heather DNA at all. This is not unexpected; an ecological study assessed honeybee foraging and found trace amounts of *C. vulgaris* DNA in honey samples harvested in May^64^. One can speculate that this is due to honey or pollen being stored in lower compartments of the hive during heather season and moved up in later months to make room for brood or to sustain colonies during periods of low flowering or adverse weather conditions.

On the other hand, there were nine out of the 234 heather honey samples that did not contain significant amounts of heather DNA ( < 0.03), with one further sample containing no heather DNA at all. These samples were collected from the Republic of Ireland (*n* = 2) and the South West (*n* = 8), where all samples from the East Midlands (*n* = 6), North East (*n* = 6), Yorkshire and the Humber (*n* = 21), Wales (*n* = 14) and Scotland (*n* = 155) contained total heather DNA as at least an important minor component.

This could be confirmed by physicochemical analyses, to see if the expected characteristics of heather honey were seen in the samples. Additionally, the chemical composition of the honeys could be evaluated by methods such as NMR^65,66^, LC-MS^67,68^, HPLC^69–71^, GC-MS^72–74^ and Raman spectroscopy^59^ to search for characteristic metabolic markers of heather. However, these tests require special instruments and more advanced analysis, often requiring extensive databases of authentic honey samples, which are challenging to produce.

Recently, government bodies have shown great interest in the application of DNA-based methodologies for food authentication, and qPCR methods have been validated for use to quantify horse and pork in processed meat products^30^. Additionally, DNA markers have been applied to detect trace DNA in rice and corn syrup adulterated honey types, with the ability to identify the presence of these syrups at only 1% adulteration level^38,75^. By applying DNA markers for different targets, the qPCR methodology could be used to simultaneously authenticate the floral sources of a honey and detect DNA indicative of sugar syrup adulteration without the need for different sample preparations or application of multiple methodologies. Moreover, our method could be multiplexed with markers for other applications, such as confirming the etymological origin of the honey bee^76^, or detecting whether genetically modified pollen is present^77,78^. Furthermore, the relative quantification analysis could be implemented with DNA markers for different botanical species to authenticate specific high-end honey types or applied to other food matrices where quantification of a particular species is beneficial or required.

In this work, an innovative qPCR method was developed for botanical authentication of heather honey with the ability to distinguish between ling (*Calluna vulgaris*) and *Erica* species of heather. Moreover, an accurate relative quantification was achieved by employing the conserved plant *trnL* P6 loop region as a plant DNA reference. The method classified 96% of 234 heather honey samples as containing ling or *Erica* heather as at least an important minor (> 3%) plant source, with the majority (68%) of samples classed as dominant (> 45%) for ling heather. Additionally, the method showed excellent specificity, with none of the 32 non-heather honey samples exceeding 1.5% amplification for either of the heather markers. The quantification produced from the qPCR method was highly comparable between melissopalynology, DNA metabarcoding and dPCR, demonstrating the suitability of real-time PCR as a robust alternative to microscopy-based pollen analysis without the need for expert analyses and interpretation. In addition, the method has high versatility and can be adapted for other floral species or used with combined markers, to simultaneously amplify multiple target DNA regions from one sample, thus reducing analysis time and costs.

## Methods

### Sample collection

266 honey samples were collected from bee farmers from regions of the UK and Ireland over three years (2021–2023). These mostly consisted of heather honey (*n* = 234) with 32 samples of non-heather honey to validate the method on honeys with a rich diversity of floral sources (Table 2, Table S1). Individual samples were further labelled by Honey type-Region-*n*, e.g., Heather-Scotland-01 (H-S-01). Samples were stored at 4 °C and away from light.

**Table 2:** Honey sample information

Honey type	Region	*n* =
Heather^a^ (H)	East Midlands (EM)	6
	North East (NE)	6
	Republic of Ireland (ROI)	2
	Scotland (S)	155
	South East (SE)	3
	South West (SW)	27
	Wales (W)	14
	Yorkshire and The Humber (Y)	21
**Total heather**		**234**
Non-heather (NH)	East (E)	4
	Northern Ireland (NI)	1
	Republic of Ireland (ROI)	1
	Scotland (S)	1
	South East (SE)	2
	South West (SW)	9
	Wales (W)	2
	West Midlands (WM)	4
	Yorkshire and The Humber (Y)	8
**Total non-heather**		**32**
**Total samples**		**266**

^a^Heather honey type indicates honeys with a suspected source of heather flowers nearby the apiaries.

Plant samples of *C. vulgaris*, *E. cinerea* and other nearby plants were purchased fresh or collected by beekeepers from their heather apiary locations and surrounding areas in 2022 and plant material was sent via post (Table 3). These plant species were tentatively identified by appearance.

**Table 3:** Plant sample information

ID^a^	species	family	common name	region
CV01	*Calluna vulgaris*	*Ericaceae*	ling heather	East
CV02	*Calluna vulgaris*	*Ericaceae*	ling heather	East
CV03	*Calluna vulgaris*	*Ericaceae*	ling heather	South West
CV04	*Calluna vulgaris*	*Ericaceae*	ling heather	South West
CV05	*Calluna vulgaris*	*Ericaceae*	ling heather	South West
CV06	*Calluna vulgaris*	*Ericaceae*	ling heather	South West
CV07^**a**^	*Calluna vulgaris*	*Ericaceae*	ling heather	Wales
CV08^**a**^	*Calluna vulgaris*	*Ericaceae*	ling heather	Wales
CV09^**a**^	*Calluna vulgaris*	*Ericaceae*	ling heather	North East
CV10^**a**^	*Calluna vulgaris*	*Ericaceae*	ling heather	South West
CV11^**a**^	*Calluna vulgaris*	*Ericaceae*	ling heather	South West
CV12^**a**^	*Calluna vulgaris*	*Ericaceae*	ling heather	Scotland
Da^**a**^	*Taraxacum spp*.	*Asteraceae*	dandelion	Scotland
EC01	*Erica cinerea*	*Ericaceae*	bell heather	South West
EC02	*Erica cinerea*	*Ericaceae*	bell heather	South West
EC03	*Erica cinerea*	*Ericaceae*	bell heather	South West
EC04	*Erica cinerea*	*Ericaceae*	bell heather	South West
EC05^**a**^	*Erica cinerea*	*Ericaceae*	bell heather	North East
EC06^**a**^	*Erica cinerea*	*Ericaceae*	bell heather	South West
EC07^**a**^	*Erica cinerea*	*Ericaceae*	bell heather	South West
EC08^**a**^	*Erica cinerea*	*Ericaceae*	bell heather	South West
ET^**a**^	*Erica tetralix*	*Ericaceae*	cross-leaved heath	South West
Mi^**a**^	*Mentha spp*.	*Lamiaceae*	mint	South West
PE^**a**^	*Potentilla erecta*	*Rosaceae*	tormentil	Scotland
PV^**a**^	*Prunella vulgaris*	*Lamiaceae*	selfheal	Scotland
RF^**a**^	*Rubus fruticosus*	*Rosaceae*	bramble	Scotland
RW^**a**^	*Chamaenerion angustifolium*	*Onagraceae*	rosebay willowherb	Scotland
SC^**a**^	*Silene coronaria*	*Caryophyllaceae*	rose campion	Scotland
SP^**a**^	*Succisa pratensis*	*Caprifoliaceae*	devil’s-bit scabious	Scotland
Th^**a**^	*Cirsium or Carduus* spp.	*Asteraceae*	thistle	Scotland

ID^**a**^ indicates a sample that was a leaf cutting (not fresh).

### Melissopalynology

Melissopalynology was used to confirm honey origin to produce honey samples that could be used as reference samples to confirm method accuracy. Four heather honey samples from different suppliers in Scotland (H-S-05), Yorkshire and The Humber (H-Y-05) and the South West (H-SW-02, H-SW-03) were sent to Minerva Scientific Ltd. (Derby, UK) for pollen analysis of floral and geographical origin. Two of these samples were thought to contain mostly ling heather (H-S-05, H-Y-05) and two bell heather or a mix of heathers (H-SW-02, H-SW-03).

Pollen analysis results were adjusted using pollen coefficient values to account for misrepresented pollen types^60,61^. Pollens with < 1% abundance were not adjusted.

### DNA extraction

The DNeasy Plant Pro kit (Qiagen, Germany) was used for DNA extraction from plant samples, with the inclusion of solution PS due to high polyphenol content in heather plant material. Plant material (≤ 100 mg) was flash frozen with liquid nitrogen and ground with a 3 mm tungsten carbide bead (Qiagen, Germany) and homogenised in the StarBeater (VWR, UK) for 2 min at 20 mHz. The powder was suspended in 900 µL buffer CD1 + 100 µL PS solution, and the homogenisation step was repeated. All following steps were completed according to the manufacturers protocol. The eluted samples were stored at −20 °C and diluted 10X in ultrapure water for PCR testing.

Honey samples were extracted using the DNeasy Plant Pro kit (Qiagen, Germany) without the inclusion of solution PS, which can reduce DNA yields when not necessary, as described in Dodd et al. (2025)^38^.

### DNA markers, PCR and qPCR

DNA sequences of relevant plants were obtained from the NIH GenBank^79^ and species-specific DNA markers were designed using Geneious Prime software (v 2022-1.1), on the *trnL* gene region (Table 4). The endogenous plant marker targeting the *trnL* P6 loop was chosen to amplify the total plant DNA in the sample, featuring highly conserved primer binding sites with a hypervariable region between them^37^. The opposite approach was taken for the construction of the species-specific markers, which were designed on hypervariable regions to obtain specificity but contained a conserved region between oligos (Fig. S1). Primer specificity was evaluated in silico using Primer-BLAST^80^ and by testing for PCR amplification on target and non-target plant DNA extracts (Table 3).

**Table 4:** Primer information

Target	Primer name	Sequence (5’-3’)	Expected product size (bp)
Conserved plant *trnL* P6 region^37^	trnL_P6_F	GGGCAATCCTGAGCCAA	10–144
	trnL_P6_R	CCATTGAGTCTCTGCACCTATC	
*Calluna vulgaris* region of *trnL*	CV_trnL_F	CATCGTTTGCTAGATCTTTTGC	98
	CV_trnL_R	CAATAAATTTCATTGTTGTCGGTC	
*Erica cinerea* region of *trnL*	EC_trnL_F	CTCCATTGTCTACTAGATCTTTTGA	101
	EC_trnL_R	CAATAAATTTCATTGTTGTCGTCA	

PCR and qPCR were run according to Dodd et al. (2025), with an annealing temperature of 60 °C^38^.

The raw C_q_ values were calculated by the CFX software (CFX Maestro Version 2.3) by applying a single threshold with baseline baseline-subtracted curve fit. The average C_q_ and standard deviation (SD) were calculated from three PCR replicates and negative controls were included in all tests.

Primer efficiency (*E*) was calculated using 5 points from a standard curve of honey extract (1X - 10,000X) diluted with ultrapure water. The obtained C_q_ values were plotted against concentration and regression analysis was applied to obtain the slope and R^2^ value. Equation (1) was used to calculate efficiency^81^.

1 $$E={10}^{(\frac{-1}{{slope}})})$$

### Digital PCR

Digital PCR (dPCR) was performed using the QIAcurity system with Software Suite 2.1.8.23 (Qiagen, Germany) with 8.5 K partitions. The cycling consisted of 95 °C for 2 min then 40 cycles of 95 °C for 15 s, 60 °C for 15 s and 72 °C for 15 s, then 40 °C for 5 min. The imaging was run with an exposure duration of 400 ms and a gain of 4. The reaction mix was performed in a 22 µL reaction using 3X QIAcurity EvaGreen Mastermix (Qiagen, Germany) with 2.2 µM forward and reverse primer and 2 µL of 10X diluted DNA extract in ultrapure water.

### Metabarcoding

PCR was performed for 30 cycles for the *trnL* P6 loop using the primer set described in Table 4, in the conditions provided above. 60 µL of PCR product was purified using the QIAquick PCR Purification Kit (Qiagen, Germany) according to manufacturer instructions. Elution was performed with 25 µL of elution buffer twice to produce 50 µL of purified product. The PCR product was sent to Novogene (Cambridge, UK) for library preparation and sequencing using Illumina paired-end 150 bp sequencing.

Quality control was performed on the raw reads using FastQC (Version 0.12.0)^82^, followed by adapter sequence trimming using Cutadapt (Version 5.0)^83^ with an error rate of 0.1. Trimmed reads were subsequently merged using VSEARCH (Version 2.29.3)^84^ and filtered to discard reads shorter than 20 bp. Dereplication and Amplicon Sequence Variants (ASV) clustering were performed using VSEARCH. Chimeric reads were subsequently removed (VSEARCH), and the resulting data were used for taxonomy assignment using BLAST (blastn)^85^. The most conservative taxonomic level was assigned by assessing all top hits with ≥ 98% sequence similarity to the ASV, and classified as species, genus or family. For example, if all top hits corresponded to the same genus but of different species, the genus was assigned.

### Relative quantification

The relative quantity of the target markers was calculated using the ${2}^{-\Delta {Ct}}$ method^86^, with the conserved plant marker used as the endogenous reference. The average C_q_ was from three technical replicates (eq. 2).

2 $$\Delta {Ct}=\left({{Average\; Cq}}_{{target}}-{{Average\; Cq}}_{{reference}}\,\right)$$

This was then corrected to ${2}^{-\mathrm{\varDelta \varDelta }{Ct}}$ using values obtained from the plant material as controls (eq. 3).

3 $$\mathrm{\varDelta \varDelta }{Ct}=\left({\Delta {Cq}}_{{sample}}-{\Delta {Cq}}_{{control}}\right)$$

The standard deviation was calculated by evaluating the ${2}^{-\Delta {Ct}}$ or ${2}^{-\mathrm{\varDelta \varDelta }{Ct}}$ plus and minus the standard deviation from the average C_q_ for the target and reference markers^86^.

For digital PCR the copies/µL was obtained and the relative quantity was calculated by Eq. (4). It was not necessary to perform technical replicates of the dPCR reaction due to the droplet formation, so calculation of standard deviation was not possible.

4 $${Relative\; quantity}({dPCR})=\frac{{{Copies}}_{{target}}}{{{Copies}}_{{reference}}}$$

## Supplementary information

botanicalDNA_supplementary1

botanicalDNA_supplementary2

botanicalDNA_supplementary3

botanicalDNA_supplementary4

## Data Availability

All data generated or analysed during this study are included in this published article and its supplementary information files. The datasets generated and analysed during the current study are available in the CORD data repository [10.57996/cran.ceres-2784].
