# Supplementary material for: Tracing the botanical origins of UK heather honey by relative quantification of plant DNA
Source: NPJ Sci Food. 2025 Sep 30;9:196. doi: 10.1038/s41538-025-00561-1 (PMC12485031; doi:10.1038/s41538-025-00561-1)
Supplement: Supplementary file 1 — botanicalDNA_supplementary1 [file 41538_2025_561_MOESM1_ESM.docx]

Tracing the botanical origins of UK heather honey by relative quantification of plant DNA.

Sophie Dodd^a^, Zoltan Kevei^a^, Zahra Karimi^a^, Jane Jennifer Sumesh Kumar^a^, Anastasios Koidis and Maria Anastasiadi^a^🖂.

^a^ Centre for Soil. Agrifood and Biosciences, Faculty of Engineering and Applied Sciences, Cranfield University, College Road, Cranfield, MK43 0AL.

^b^Institute for Global Food Security, Queen’s University of Belfast, Belfast, BT9 5BN.

🖂 Corresponding authors: [m.anastasiadi@cranfield.ac.uk](mailto:m.anastasiadi@cranfield.ac.uk)

**Supplementary Data 1**

**Figure S1**: Position of oligos on aligned *Calluna vulgaris* (1.) and *Erica cinerea* (2.) *trnL* sequences (screenshot from Genious Prime software).

**<-----------------tRNA-Leu (trnL) gene region--------------------->**


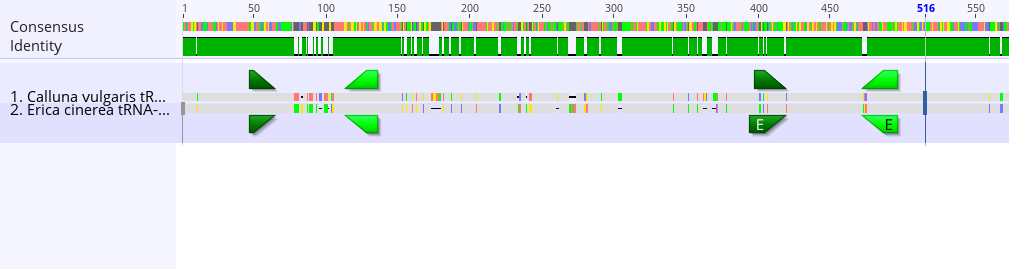


**--specific marker--**

(Our design)

(Taberlet et al., 2007)

**--P6 loop--**


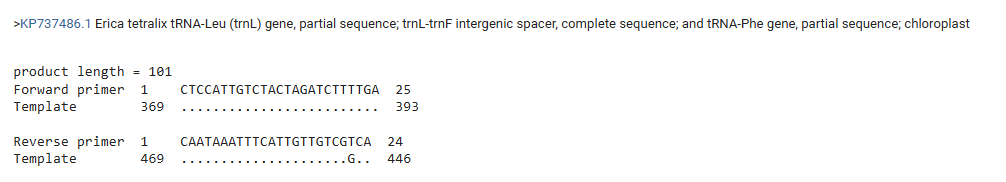


**Figure S2:** PrimerBLAST result of the *Erica cinerea* EC_trnL marker showing the mismatch on the reverse primer with *Erica teralix*.

**Table S1: Details of 15 non-heather honey samples from diverse floral sources**

| **Sample #** | **Honey description** | **Bee forage *** | **Season** |
| --- | --- | --- | --- |
| NH-Y-01 | woodland | Woodland trees and nearby flowers, including lime (*Tilia*), horse chestnut (*Aesculus hippocastanum)* and sweet chestnut (*Castanea sativa*). | Summer |
| NH-Y-02 | sycamore | Predominantly *Acer pseudoplatanus* with a bit of hawthorn (*Crataegus monogyna)* and bean (*Fabaceae*) | Spring |
| NH-Y-03 | phacelia | *Phacelia tanacetifolia* | Spring |
| NH-Y-04 | ivy | *Hedera helix* | Autumn |
| NH-Y-05 | Himalayan balsam | *Impatiens glandulifera* | Autumn |
| NH-Y-06 | spring set | Mixture of farmed and wild spring flowers | Spring |
| NH-WM-01 | borage | *Borago officinalis* | Summer |
| NH-Y-07 | buckwheat | *Fagopyrum esculentum* | Autumn |
| NH-WM-02 | meadowfoam | *Limnanthes alba* | Summer |
| NH-E-01 | sea lavender | *Limonium vulgare* | Summer |
| NH-WM-03 | echium | *Echium plantagineum* | Summer |
| NH-E-02 | hedgerow | Mixture of flowers from hedgerows, meadows, and farmland. | Mixed |
| NH-Y-08 | English blossom | Blend of blossoms from spring and summer | Mixed |
| NH-E-03 | apple blossom | *Malus domestica* | Spring |
| NH-WM-04 | wildflower | Mixture of summer wildflowers | Summer |

* As described by honey producer

**Table S2**: Amplification (Cq) of target plant extracts with the Calluna vulgaris (CV_trnL) and Erica cinerea (EC_trnL) specific markers, where the Cq for the target marker is highlighted in bold. The average Cq and standard deviation (SD) are shown from three technical replicates.

| Sample | CV_trnL | | EC_trnL | |
| --- | --- | --- | --- | --- |
|  | C_q_ | SD | C_q_ | SD |
| CV01 | **16.41** | 0.08 | 37.13 | 0.97 |
| CV02 | **15.84** | 0.04 | 36.49 | 0.42 |
| CV03 | **15.68** | 0.06 | 35.15 | 1.37 |
| CV04 | **14.99** | 0.06 | 36.05 | 1.70 |
| CV05 | **16.50** | 0.02 | 33.75 | 0.79 |
| CV06 | **20.33** | 0.05 | ^b^ | ^b^ |
| CV07^a^ | **21.10** | 0.17 | ^b^ | ^b^ |
| CV08 ^a^ | **20.42** | 0.05 | ^b^ | ^b^ |
| CV09 ^a^ | **19.83** | 0.11 | ^b^ | ^b^ |
| CV10 ^a^ | **19.38** | 0.04 | ^b^ | ^b^ |
| CV11 ^a^ | **22.51** | 0.22 | ^b^ | ^b^ |
| CV12 ^a^ | **17.37** | 0.02 | 33.52 | 1.6 |
| EC01 | 25.46 | 0.07 | **14.44** | 0.01 |
| EC02 | 26.86 | 0.22 | **15.25** | 0.02 |
| EC03 | 28.30 | 0.14 | **17.21** | 0.10 |
| EC04 | 36.55 | 0.36 | **18.21** | 0.30 |
| EC05 ^a^ | 30.63 | 0.67 | **17.17** | 0.07 |
| EC06 ^a^ | ^b^ | ^b^ | **20.27** | 0.08 |
| EC07 ^a^ | ^b^ | ^b^ | **18.77** | 0.14 |
| EC08 ^a^ | ^b^ | ^b^ | **19.60** | 0.07 |

^a^plant cutting ^b^no amplification

| Sample | trnL_P6 | | CV_trnL | | EC_trnL |  |
| --- | --- | --- | --- | --- | --- | --- |
|  | C_q_ | SD | C_q_ | SD | Cq | SD |
| CV05 | 20.41 | 0.07 | 20.15 | 0.10 | 37.63 | 3.24 |
| EC01 | 21.16 | 0.11 | 31.90 | 0.18 | 20.90 | 0.03 |
| RW | 17.12 | 0.39 | 37.13 | 1.73 | 35.04 | 0.92 |
| Th | 15.93 | 0.30 | 35.69 | 1.11 | 34.96 | 0.32 |
| ET | 16.96 | 0.32 | ^a^ | ^a^ | 17.55 | 0.25 |
| Mi | 16.11 | 0.04 | 33.64 | 0.64 | 34.97 | 1.14 |
| PE | 14.98 | 0.40 | ^a^ | ^a^ | ^a^ | ^a^ |
| PV | 15.60 | 0.05 | ^a^ | ^a^ | ^a^ | ^a^ |
| RF | 16.31 | 0.11 | 36.02 | 1.02 | 35.83 | 0.26 |
| SC | 16.17 | 0.37 | 34.55 | 0.30 | 35.65 | 0.45 |
| SP | 16.25 | 0.46 | 36.52 | 1.42 | 35.62 | 2.13 |
| Da | 17.24 | 0.32 | 33.46 | 0.57 | 34.82 | 0.91 |

**Table S3**: Amplification (C_q_) of plant extracts with plant (trnL_P6), *Calluna vulgaris* (CV_trnL) and *Erica cinerea* (EC_trnL) markers.

^a^no amplification

**Table S4**: Relative quantity (RQ) of the ling (*Calluna vulgaris*) and bell (*Erica spp.)* marker in honey extracts amplified with qPCR, grouped by honey type and region. For each group the RQ mean, range and standard deviation (SD) are shown.

| **Honey type** | **Region** | ***n*** | ***Calluna vulgaris* RQ** | | | | ***Erica* spp. RQ** | | | |
| --- | --- | --- | --- | --- | --- | --- | --- | --- | --- | --- |
|  |  |  | **mean** | **min** | **max** | **SD** | **mean** | **min** | **max** | **SD** |
| Heather^a^ (H) | EM | 6 | 0.62 | 0.39 | 1.02 | 0.23 | 0.00 | 0.00 | 0.00 | 0.00 |
|  | NE | 5 | 0.94 | 0.55 | 1.48 | 0.36 | 0.02 | 0.00 | 0.09 | 0.04 |
|  | ROI | 2 | 0.02 | 0.00 | 0.03 | 0.02 | 0.00 | 0.00 | 0.00 | 0.00 |
|  | S | 155 | 0.64 | 0.04 | 1.67 | 0.24 | 0.02 | 0.00 | 0.42 | 0.04 |
|  | SE | 3 | 0.71 | 0.60 | 0.89 | 0.16 | 0.03 | 0.01 | 0.05 | 0.02 |
|  | SW | 27 | 0.17 | 0.00 | 0.93 | 0.29 | 0.08 | 0.00 | 0.36 | 0.10 |
|  | W | 14 | 0.49 | 0.08 | 0.95 | 0.25 | 0.08 | 0.00 | 0.50 | 0.15 |
|  | Y | 21 | 0.54 | 0.01 | 1.24 | 0.37 | 0.02 | 0.00 | 0.14 | 0.04 |
| **Total heather** | | **234** | **0.57** | **0.00** | **1.67** | **0.31** | **0.03** | **0.00** | **0.50** | **0.07** |
| Non-heather (NH) | E | 4 | 0.00 | 0.00 | 0.00 | 0.00 | 0.00 | 0.00 | 0.00 | 0.00 |
|  | NI | 1 | 0.00 | 0.00 | 0.00 | ^b^ | 0.00 | 0.00 | 0.00 | ^b^ |
|  | ROI | 1 | 0.00 | 0.00 | 0.00 | ^b^ | 0.00 | 0.00 | 0.00 | ^b^ |
|  | S | 1 | 0.00 | 0.00 | 0.00 | ^b^ | 0.00 | 0.00 | 0.00 | ^b^ |
|  | SE | 2 | 0.00 | 0.00 | 0.00 | 0.00 | 0.00 | 0.00 | 0.00 | 0.00 |
|  | SW | 9 | 0.00 | 0.00 | 0.01 | 0.00 | 0.00 | 0.00 | 0.02 | 0.01 |
|  | W | 2 | 0.00 | 0.00 | 0.01 | 0.00 | 0.01 | 0.00 | 0.02 | 0.01 |
|  | WM | 4 | 0.01 | 0.00 | 0.02 | 0.01 | 0.00 | 0.00 | 0.00 | 0.00 |
|  | Y | 8 | 0.00 | 0.00 | 0.01 | 0.00 | 0.00 | 0.00 | 0.00 | 0.00 |
| **Total non-heather** | | **32** | **0.00** | **0.00** | **0.02** | **0.00** | **0.00** | **0.00** | **0.02** | **0.01** |

^a^Heather honey type indicates honeys with a suspected source of heather flowers nearby the apiaries ^b^SD was not applicable as group n = 1

**Table S5**: Comparison of the quantification of *Calluna vulgaris* and *Erica* spp pollen from melissopalynology, metabarcoding, qPCR and dPCR.

| sample | ***Calluna vulgaris* abundance** | | | | ***Erica spp* abundance** | | | |
| --- | --- | --- | --- | --- | --- | --- | --- | --- |
|  | pollen | metabarcoding | qPCR*^a^* | dPCR*^a^* | pollen | metabarcoding | qPCR*^a^* | dPCR*^a^* |
| H-S-05 | 31% | 47% | 61% | 52% | *^b^* | *^b^* | *^b^* | *^b^* |
| H-Y-05 | 60% | 58% | 55% | 45% | *^b^* | *^b^* | *^b^* | 2% |
| H-SW-02 | 4% | >1%^a^ | 0.001% | 0.1% | >1%^a^ | 7% | 1% | 1% |
| H-SW-03 | >1%^a^ | >1%^a^ | 0.2% | 1% | 9% | 9% | 9% | 10% |

*^a^*pollen coefficient correction not applied ^b^not detected
