# Supplementary material for: Tracing the botanical origins of UK heather honey by relative quantification of plant DNA
Source: NPJ Sci Food. 2025 Sep 30;9:196. doi: 10.1038/s41538-025-00561-1 (PMC12485031; doi:10.1038/s41538-025-00561-1)
Supplement: Supplementary file 2 — botanicalDNA_supplementary2 [file 41538_2025_561_MOESM2_ESM.docx]

Tracing the botanical origins of UK heather honey by relative quantification of plant DNA.

Sophie Dodd^a^, Zoltan Kevei^a^, Zahra Karimi^a^, Jane Jennifer Sumesh Kumar^a^, Anastasios Koidis and Maria Anastasiadi^a^*.

^a^ Centre for Soil. Agrifood and Biosciences, Faculty of Engineering and Applied Sciences, Cranfield University, College Road, Cranfield, MK43 0AL.

^b^Institute for Global Food Security, Queen’s University of Belfast, Belfast, BT9 5BN.

* Corresponding authors: [m.anastasiadi@cranfield.ac.uk](mailto:m.anastasiadi@cranfield.ac.uk)

**Supplementary Data 2**

**Supplementary Data 2: *in silico* primer specificity testing of *Calluna vulgaris* marker CV_trnL using primer-BLAST**

PrimerBLAST search: nt database; plants (taxid 3193); shows all entries with up to 6 mismatches to primer pair and up to 2 mismatches in last 5 bp at 3’ ends.

**Summary table**

| **No. mismatches to primers (F; R)** | **No. Species and Family.** | **Species (no. isolate matches in database).** |
| --- | --- | --- |
| 0 ; 0 | No. species: 1  Family: Ericaceae  (*Calluna* = 1). | Calluna vulgaris (211) |
| 2 ; 1 | No. species: 9  Family: Ericaceae (*Rhodedendron* = 3, Menziesia = 3, *Empetrum* = 1, *Coremia* = 1, *Daboecia* = 1, *Kalmia* = 1) | Empetrum nigrum (26), Corema album (2), Daboecia cantabrica (2), Kalmia procumbens (1), Rhododendron camtschaticum (4), Menziesia multiflora (1), Menziesia katsumatae (1), Menziesia goyozanensis (1), Rhododendron benhallii (1). |
| 3 ; 1 | No. species: 171  Family: Ericaceae (*Rhododendron* = 165, *Kalmia* = 4, *Menziesia* = 2) | Rhododendron delavayi (25), Rhododendron ferrugineum (6), Rhododendron nivale (1), Rhododendron rupicola (2), Rhododendron phaeochrysum (2), Rhododendron aganniphum (1), Rhododendron arboreum (96), Kalmia procumbens (23), Rhododendron sp. (36), Rhododendron decorum (18), Rhododendron williamsianum (4), Rhododendron mucronulatum (4), Rhododendron sichotense (2), Rhododendron dauricum (1), Rhododendron ledebourii (2), Rhododendron purdomii (1), Rhododendron ambiguum (2), Rhododendron przewalskii (2), Rhododendron stapfianum (1), Rhododendron fallacinum (8), Rhododendron planecostatum (1), Rhododendron crassifolium (5), Rhododendron buxifolium (4), Rhododendron rugosum (5), Rhododendron exuberans (1), Rhododendron lowii (2), Rhododendron suaveolens (5) , Rhododendron stenophyllum (3), Rhododendron cuneifolium (2), Rhododendron praetervisum (2), Rhododendron javanicum (4), Rhododendron orbiculatum (2), Rhododendron borneense (1), Rhododendron acuminatum (1), Rhododendron bagobonum (1), Rhododendron anthopogonoides (1), Rhododendron capitatum (1), Rhododendron thymifolium (1), Rhododendron micranthum (4), Rhododendron calophytum (2), Rhododendron oreodoxa (1), Rhododendron minus (11), Rhododendron carolinianum (4), Rhododendron chapmanii (1), Rhododendron smokianum (2), Rhododendron shanii (1), Rhododendron duclouxii (12), Rhododendron spinuliferum (8), Rhododendron spiciferum (10), Rhododendron scabrifolium (5), Rhododendron mollicomum (1), Rhododendron virgatum (1), Kalmia angustifolia (2), Rhododendron emarginatum (1), Rhododendron himantodes (1), Rhododendron loranthiflorum (1), Rhododendron edanoi (1), Rhododendron konori (1), Rhododendron polyanthemum (1), Rhododendron macgregoriae (1), Rhododendron lochiae (1), Rhododendron laetum (1), Rhododendron aurigeranum (1), Rhododendron retusum (1), Rhododendron zaleucum (1), Rhododendron lutescens (1), Rhododendron christianae (1), Rhododendron xanthostephanum (1), Rhododendron auritum (1), Rhododendron racemosum (2), Rhododendron hirsutum (1), Rhododendron moupinense (1), Rhododendron tomentosum (2), Rhododendron tapetiforme (1), Rhododendron setosum (1), Rhododendron polycladum (1), Rhododendron nitidulum (1), Rhododendron hippophaeoides (1), Rhododendron fastigiatum (2), Rhododendron rubiginosum (1), Rhododendron bracteatum (1), Rhododendron charitopes (1), Rhododendron keysii (1), Rhododendron cinnabarinum (1), Rhododendron leucaspis (1), Rhododendron schlippenbachii (5), Rhododendron pilosum (1), Rhododendron albrechtii (3), Rhododendron vaseyi (1), Rhododendron prunifolium (1), Rhododendron prinophyllum (1), Rhododendron periclymenoides (1), Rhododendron occidentale (3), Rhododendron cumberlandense (1), Rhododendron austrinum (1), Rhododendron atlanticum (3), Rhododendron arborescens (1), Rhododendron sherriffii (1), Rhododendron alutaceum (1), Rhododendron selense (2), Rhododendron smirnowii (2), Rhododendron ponticum (4), Rhododendron macrophyllum (2), Rhododendron degronianum (3), Rhododendron brachycarpum (3), Rhododendron elliottii (1), Rhododendron neriiflorum (3), Rhododendron strigillosum (2), Rhododendron annae (1), Rhododendron griersonianum (3), Rhododendron protistum (1), Rhododendron macabeanum (2), Rhododendron campylocarpum (2), Rhododendron auriculatum (1), Rhododendron thayerianum (1), Rhododendron ririei (1), Rhododendron albiflorum (2), Kalmia buxifolia (3), Rhododendron concinnum (1), Rhododendron henanense (1), Rhododendron molle (1), Rhododendron aureum (6), Rhododendron kawakamii (1), Rhododendron datiandingense (1), Rhododendron platypodum (1), Rhododendron cyanocarpum (2), Rhododendron lapponicum (2), Rhododendron groenlandicum (1), Rhododendron irroratum (2), Rhododendron insigne (1), Rhododendron eclecteum (1), Rhododendron taliense (1), Rhododendron forrestii (1), Rhododendron mallotum (1), Rhododendron floccigerum (1), Rhododendron catacosmum (1), Rhododendron beanianum (1), Rhododendron pseudochrysanthum (1), Rhododendron anhweiense (1), Rhododendron tsariense (1), Rhododendron mengtszense (1), Rhododendron aberconwayi (2), Rhododendron grande (1), Rhododendron fulvum (1), Rhododendron praevernum (1), Rhododendron orbiculare (1), Rhododendron rothschildii (1), Rhododendron wardii (1), Rhododendron campanulatum (1), Rhododendron barbatum (1), Rhododendron argyrophyllum (1), Rhododendron coryanum (1), Rhododendron adenopodum (1), Rhododendron lanigerum (1), Rhododendron agastum (1), Rhododendron maximum (2), Rhododendron hybrid (4), Kalmia latifolia (3), Rhododendron sinogrande (1), Rhododendron rex (1), Rhododendron edgeworthii (1), Rhododendron dimitrum (1), Rhododendron anthosphaerum (1), Rhododendron hyperythrum (1), Rhododendron ungernii (1), Rhododendron caucasicum (1), Rhododendron catawbiense (1), Menziesia pilosa (2), Menziesia ferruginea (1), Rhododendron quinquefolium (2), Rhododendron pentaphyllum (2) |
| 2 ; 2 | No. species: 2  Family: Zingiberaceae (*n* = 2) | Siphonochilus kirkii (4), Siphonochilus decorus (3), |
| 1 ; 3 | No. species: 1  Family: Ericaceae (*Harrimanella* = 1) | Harrimanella hypnoides (1), |
| 3 ; 2 | No. species: 297  Family: Theaceae (*n* = 112), Sapotaceae (*n* = 104), Bromeliaceae (*n* = 39),  Orchidaceae (*n* = 14), Fabaceae (*n* = 12), Ericaceae (*Rhododendron* = 2, *Bejaria* = 1), Asteliaceae (*n* = 3), Zingiberaceae (*n* = 3), Olacaceae (*n* = 2), , Proteaceae (*n* = 2), Clethraceae (*n* = 1), Loasaceae (*n* = 1), Euphorbiaceae (*n* = 1). | Rhododendron pachypodum (1), Bulbophyllum mentosum (1), Senna obtusifolia (3), Camellia mingii (1), Astelia australiana (2), Manilkara zapota (3), Camellia weiningensis (1), Manilkara mochisia (1), Pradosia ptychandra (4), Pouteria rodriguesiana (2), Pouteria guianensis (4), Pouteria gongrijpii (5), Pouteria eugeniifolia (2), Pouteria egregia (2), Pouteria decorticans (1), Pouteria cladantha (1), Micropholis melinoniana (1), Micropholis guyanensis (8), Micropholis cayennensis (1), Manilkara huberi (1), Ragala sanguinolenta (2), Chrysophyllum prieurii (2), Chrysophyllum lucentifolium (1), Chrysophyllum cuneifolium (2), Caperonia stenophylla (1), Xantolis weimingii (1), Sideroxylon spinosum (3), Camellia costata (18), Senna rugosa (1), Psychopsis sanderae (2), Astelia alpina (2), Camellia melliana (1), Camellia tachangensis (17), Camellia pilosperma (1), Camellia gymnogyna (6), Camellia fangchengensis (5), Camellia quinquelocularis (1), Camellia impressinervis (2), Camellia cuspidate (1), Manilkara kauki (1), Camellia sinensis (8), Camellia sp. (5), Camellia pitardii (5), Camellia paucipetala (1), Camellia longistyla (1), Camellia kweichowensis (1), Camellia delicata (1), Phanerodiscus capuronii (2), Camellia reticulata (8), Camellia semiserrata (7), Camellia saluenensis (2), Camellia hongkongensis (1), Stewartia obovata (1), Stewartia micrantha (1), Hartia laotica (1), Stewartia crassifolia (3), Stewartia cordifolia (3), Stewartia sinii (3), Stewartia pteropetiolata (7), Stewartia villosa (3), Schima superba (13), Stewartia sinensis (11), Stewartia pseudocamellia (8), Stewartia malacodendron (6), Stewartia monadelpha (4), Stewartia ovata (9), Stewartia rostrata (4), Stewartia rubiginosa (2), Stewartia serrata (2), Palaquium sp. (3), Madhuca dubardii (2), Sideroxylon lanuginosum (1), Sideroxylon wightianum (1), Astelia pumila (6), Senna tora (4), Cryptochilus luteus (1), Eria siamensis (1), Cryptochilus strictus (1), Camellia euphlebia (2), Blumenbachia dissecta (1), Pachites bodkinii (3), Pachites appressus (1), Gordonia lasianthus (3), Schima argentea (8), Stewartia calcicole (1), Franklinia alatamaha (4), Camellia mairei (1), Gordonia brandegeei (1), Schima wallichii (11), Schima sinensis (8), Schima sericans (5), Schima remotiserrata (3), Schima noronhae (3), Schima multibracteata (1), Schima khasiana (7), Schima crenata (1), Schima brevipedicellata (4), Camellia salicifolia (2), Camellia liberistyloides (1), Camellia grijsii (2), Camellia danzaiensis (4), Camellia leptophylla (3), Camellia ptilophylla (3), Camellia pubescens (3), Camellia arborescens (2), Camellia kwangsiensis (3), Camellia huana (2), Pouteria campechiana (2), Camellia albosericea (1), Camellia pingguoensis (5), Camellia micrantha (3), Camellia pubipetala (3), Camellia wumingensis (2), Cephalanthera humilis (1), Senna reticulata (1), Pouteria splendens (1), Aechmea andersoniana (1), Hohenbergia portoricensis (2), Hohenbergia spinulosa (2), Aechmea amorimii (1), Hohenbergia penduliflora (2), Hohenbergia proctorii (1), Hohenbergia antillana (1), Aechmea patentissima (2), Aechmea sulbahianensis (1), Aechmea lingulatoides (3), Hohenbergia distans (1), Hohenbergia jamaicana (1), Aechmea turbinocalyx (1), Ronnbergia brasiliensis (2), Aechmea pernambucentris (1), Aechmea bicolor (1), Hohenbergia inermis (1), Hohenbergia mesoamericana (1), Aechmea froesii (1), Hohenbergia caymanensis (1), Hohenbergia sp. (1), Aechmea maranguapensis (1), Aechmea incompta (1), Aechmea linharesiorum (1),  Camellia magniflora (1), Camellia bailinshanica (1), Viridantha plumosa (2), Camellia omeiensis (2), Camellia oligophlebia (1), Camellia jinshajiangica (1), Camellia borealiyunnanica (2), Camellia brevipetiolata (1), Camellia lungshenensis (2), Camellia caudata (2), Camellia ptilosperma (2), Stewartia sichuanensis (1), Camellia minima (1), Camellia insularis (1), Pouteria viridis (1), Camellia nitidissima (15), Camellia longzhouensis (1), Camellia chrysanthoides (5), Camellia flavida (1), Camellia fascicularis (2), Camellia parvipetala (2), Camellia lienshanensis (1), Camellia bambusifolia (1), Schima parviflora (2), Schima sp. (1), Sophora sp. (1), Mimusops elengi (2), Camellia phellocapsa (1), Camellia macrosepala (1), Camellia limonia (2), Camellia hekouensis (1), Camellia euryoides (1), Camellia angustifolia (1), Camellia acutissima (1), Sapotaceae sp. (2), Ormosia bancana (1), Tainia acuminata (1), Camellia polyodonta (1), Camellia indochinensis (1), Rhododendron argyrophyllum (1), Mimusops coriacea (1), Astelia fragrans (1), Earina autumnalis (2), Banksia plumosa (1), Banksia carlinoides (1), Canistrum aurantiacum (1), Billbergia tweedieana (1), Pouteria xerocarpa (1), Planchonella myrsinodendron (1), Niemeyera prunifera (2), Tieghemella heckelii (1), Tieghemella Africana (1), Baillonella toxisperma (1), Autranella congolensis (1), Pouteria aningeri (1), Gambeya subnuda (1), Gambeya lacourtiana (1), Gambeya gigantea (1), Gambeya Africana (1), Astelia nervosa (2), Camellia perpetua (2), Planchonella cinerea (1), Schima mertensiana (2), Camellia rostrata (1), Faucherea thouvenotii (1), Mimusops capuronii (1), Capurodendron madagascariense (1), Olax sp. (1), Camellia debaoensis (1), Synsepalum dulcificum (3), Camellia crapnelliana (1), Epipactis helleborine (1), Myrocarpus fastigiatus (1), Collospermum microspermum (1), Collospermum hastatum (2), Astelia nadeaudii (1), Astelia papuana (1), Astelia neocaledonica (1), Astelia menziesiana (2), Astelia argyrocoma (1), Astelia graminea (1), Astelia banksia (2), Pouteria caimito (2), Vitellaria paradoxa (2), Madhuca hainanensis (1), Camellia fraternal (1), Camellia chuongtsoensis (1), Sideroxylon cinereum (1), Stewartia tonkinensis (2), Stewartia laotica (1), Hohenbergia negrilensis (1), Senna gardneri (1), Pouteria singularis (1), Gomesa radicans (1), Xantolis siamensis (1), Van-royena castanosperma (1), Synsepalum passargei (1), Sarcaulus brasiliensis (1), Pradosia surinamensis (1), Pradosia brevipes (1), Pouteria vernicosa (1), Sersalisia sericea (1), Pouteria multiflora (1), Pouteria macrophylla (1), Planchonella linggensis (1), Planchonella howeana (1), Pouteria hispida (1), Pouteria gardneriana (1), Micropholis egensis (1), Pouteria domingensis (1), Pouteria alnifolia (1), Planchonella kaalaensis (1), Pichonia daenikeri (1), Pichonia balansana (1), Omphalocarpum pachysteloides (1), Pycnandra francii (1), Amorphospermum antilogum (1), Neohemsleya usambarensis (1), Micropholis venulose (1), Leptostylis filipes (1), Planchonella baillonii (1), Englerophytum natalense (1), Englerophytum magalismontanum (1), Elaeoluma schomburgkiana (1), Ecclinusa ramiflora (1), Ecclinusa guianensis (1), Diploon cuspidatum (1), Delpydora macrophylla (1), Delpydora gracilis (1), Chrysophyllum venezuelanense (1), Chrysophyllum fenerivense (1), Chrysophyllum pruniforme (1), Chrysophyllum boivinianum (1), Chrysophyllum bangweolense (1), Breviea sericea (1), Pleioluma sebertii (1), Pleioluma baueri (1), Aubregrinia taiensis (1), Elaeoluma glabrescens (1), Chrysophyllum imperial (1), Chromolucuma rubriflora (1), Swartzia panacoco (1), Swartzia myrtifolia (1), Swartzia acutifolia (1), Bulbophyllum aubrevillei (1), Madhuca macrophylla (1), Argania spinosa (1), Aulotandra trialata (1), Aulotandra cf. trigonocarpa (3), Siphonochilus aethiopicus (1), Stewartia gemmate (1), Isotropis forrestii (2), Bejaria aestuans (1), Eberhardtia tonkinensis (1), Clethra fabri (1), Psychopsis papilio (2), Planchonella novozelandica (1), |
| 2 ; 3 | No. species: 5  Family: Campanulaceae (*n* = 5) | Codonopsis atriplicifolia (2), Codonopsis javanica (1), Cyananthus lobatus (1), Codonopsis bhutanica (1), Leptocodon hirsutus (1), |
| 4 ; 2 | No. species: 5  Family: Orchidaceae (*n* = 4), Bromeliaceae (*n* = 1). | Tainia dunnii (2), Tainia cordifolia (4), Hohenbergia laesslei (1), Tainia sp. (1), Tainia macrantha (1), |
| 3 ; 3 | No. species: 11  Family:  Orchidaceae (*n* = 4),  Ericaceae (*Vaccinium* = 2), Betulaceae (*n* = 1), Fabaceae (*n* = 1), Campanulaceae (*n* = 1),  Bromeliaceae (*n* = 1),  Solanaceae (*n* = 1). | Eria coronaria (1), Mesoamerantha guatemalensis (1), Zygostates apiculate (1), Zygostates grandiflora (1), Zygostates alleniana (1), Solanum cheesmaniae (1), Vaccinium oxycoccos (3), Vaccinium microcarpum (2), Alnus incana (1), Chamaecrista ericifolia (1), Cyclocodon axillaris (1), |

**Primer-BLAST results for *Calluna vulgaris* marker with multiple isolates removed. Top 10 results shown, full analysis available on CORD (doi).**

>[OZ071054.1](https://www.ncbi.nlm.nih.gov/nucleotide/2739979359?from=24233&to=24330&report=gbwithparts) Calluna vulgaris genome assembly, organelle: plastid:chloroplast

product length = 98

Forward primer 1 CATCGTTTGCTAGATCTTTTGC 22

Template 24330 ...................... 24309

Reverse primer 1 CAATAAATTTCATTGTTGTCGGTC 24

Template 24233 ........................ 24256

>[LT606490.1](https://www.ncbi.nlm.nih.gov/entrez/viewer.fcgi?db=nucleotide&id=1550055805) Empetrum nigrum chloroplast genomic DNA containing trnL(UAA) intron region, isolate L600-2-8 - Sequence 4

product length = 97

Forward primer 1 CATCGTTTGCTAGATCTTTTGC 22

Template 307 ......C..............A 328

Reverse primer 1 CAATAAATTTCATTGTTGTCGGTC 24

Template 403 .......................A 380

>[OZ076487.1](https://www.ncbi.nlm.nih.gov/entrez/viewer.fcgi?db=nucleotide&id=2745680740) Corema album genome assembly, organelle: plastid:chloroplast

product length = 98

Forward primer 1 CATCGTTTGCTAGATCTTTTGC 22

Template 159880 ......C..............A 159859

Reverse primer 1 CAATAAATTTCATTGTTGTCGGTC 24

Template 159783 .......................A 159806

>[KP737379.1](https://www.ncbi.nlm.nih.gov/entrez/viewer.fcgi?db=nucleotide&id=815932620) Daboecia cantabrica tRNA-Leu (trnL) gene, partial sequence; trnL-trnF intergenic spacer, complete sequence; and tRNA-Phe gene, partial sequence; chloroplast

product length = 98

Forward primer 1 CATCGTTTGCTAGATCTTTTGC 22

Template 387 ......C..............A 408

Reverse primer 1 CAATAAATTTCATTGTTGTCGGTC 24

Template 484 .......................A 461

>[MT779562.1](https://www.ncbi.nlm.nih.gov/entrez/viewer.fcgi?db=nucleotide&id=2100348563) Kalmia procumbens voucher OLD06733 trnL-trnF intergenic spacer region, partial sequence; plastid

product length = 98

Forward primer 1 CATCGTTTGCTAGATCTTTTGC 22

Template 359 ......C..............A 380

Reverse primer 1 CAATAAATTTCATTGTTGTCGGTC 24

Template 456 .......................A 433

>[MT779552.1](https://www.ncbi.nlm.nih.gov/entrez/viewer.fcgi?db=nucleotide&id=2100348553) Rhododendron camtschaticum voucher 100317 trnL-trnF intergenic spacer region, partial sequence; plastid

product length = 98

Forward primer 1 CATCGTTTGCTAGATCTTTTGC 22

Template 357 ......C..............A 378

Reverse primer 1 CAATAAATTTCATTGTTGTCGGTC 24

Template 454 .......................A 431

>[AB080038.1](https://www.ncbi.nlm.nih.gov/entrez/viewer.fcgi?db=nucleotide&id=22324271) Menziesia multiflora chloroplast trnL gene, intron sequence

product length = 98

Forward primer 1 CATCGTTTGCTAGATCTTTTGC 22

Template 406 ........A............A 427

Reverse primer 1 CAATAAATTTCATTGTTGTCGGTC 24

Template 503 .......................A 480

>[AB080037.1](https://www.ncbi.nlm.nih.gov/entrez/viewer.fcgi?db=nucleotide&id=22324270) Menziesia katsumatae chloroplast trnL gene, intron sequence

product length = 98

Forward primer 1 CATCGTTTGCTAGATCTTTTGC 22

Template 406 ........A............A 427

Reverse primer 1 CAATAAATTTCATTGTTGTCGGTC 24

Template 503 .......................A 480

>[AB080036.1](https://www.ncbi.nlm.nih.gov/entrez/viewer.fcgi?db=nucleotide&id=22324269) Menziesia goyozanensis chloroplast trnL gene, intron sequence

product length = 98

Forward primer 1 CATCGTTTGCTAGATCTTTTGC 22

Template 406 ........A............A 427

Reverse primer 1 CAATAAATTTCATTGTTGTCGGTC 24

Template 503 .......................A 480

>[AB080034.1](https://www.ncbi.nlm.nih.gov/entrez/viewer.fcgi?db=nucleotide&id=22324267) Rhododendron benhallii chloroplast trnL gene, intron sequence

product length = 98

Forward primer 1 CATCGTTTGCTAGATCTTTTGC 22

Template 406 ........A............A 427

Reverse primer 1 CAATAAATTTCATTGTTGTCGGTC 24

Template 503 .......................A 480
