# Supplementary material for: Tracing the botanical origins of UK heather honey by relative quantification of plant DNA
Source: NPJ Sci Food. 2025 Sep 30;9:196. doi: 10.1038/s41538-025-00561-1 (PMC12485031; doi:10.1038/s41538-025-00561-1)
Supplement: Supplementary file 3 — botanicalDNA_supplementary3 [file 41538_2025_561_MOESM3_ESM.docx]

Tracing the botanical origins of UK heather honey by relative quantification of plant DNA.

Sophie Dodd^a^, Zoltan Kevei^a^, Zahra Karimi^a^, Jane Jennifer Sumesh Kumar^a^, Anastasios Koidis and Maria Anastasiadi^a^*.

^a^ Centre for Soil. Agrifood and Biosciences, Faculty of Engineering and Applied Sciences, Cranfield University, College Road, Cranfield, MK43 0AL.

^b^Institute for Global Food Security, Queen’s University of Belfast, Belfast, BT9 5BN.

* Corresponding authors: [m.anastasiadi@cranfield.ac.uk](mailto:m.anastasiadi@cranfield.ac.uk)

**Supplementary Data 3**

**Supplementary Data 3: *in silico* primer specificity testing of bell marker EC_trnL using primer-BLAST**

Primer-BLAST search: nt database; plants (taxid 3193); shows all entries with up to 6 mismatches to primer pair and up to 2 mismatches in last 5 bp at 3’ ends.

**Summary table**

| **No. mismatches to primers (F; R).** | **No. Species and Family.** | **Species (no. isolate matches in database).** |
| --- | --- | --- |
| 0 ; 0 | No. species: 1  Family: Ericaceae  (*Erica* = 1). | Erica cinerea (4) |
| 0 ; 1 | No. species: 155  Family: Ericaceae  (*Erica* = 155). | Erica mannii (3), Erica viscaria (2), Erica stokoei (1), Erica phaeocarpa (1), Erica parilis (1), Erica nevillei (1), Erica situshiemalis (1), Erica walkeria (1), Erica venustiflora (1), Erica sonderiana (1), Erica placentiflora (2), Erica penicilliformis (1), Erica paludicola (1), Erica lowryensis (1), Erica lasciva (2), Erica imbricata (6), Erica dracomontana (1), Erica coccinea (2), Erica cerinthoides (3), Erica woodii (1), Erica villosa (1), Erica vestita (2), Erica verticillate (1), Erica verecunda (1), Erica velatiflora (1), Erica vallis-aranearum (1), Erica uysii (1), Erica urceolata (1), Erica sp. (1), Erica tysonii (1), Erica tristis (1), Erica toringbergensis (1), Erica thunbergii (1), Erica tenuis (2), Erica stylaris (1), Erica strigose (1), Erica strigilifolia (1), Erica sparsa (1), Erica sparrmannii (1), Erica simulans (1), Erica sylvatica (1), Erica serrata (1), Erica rivularis (1), Erica rigidula (1), Erica rhopalantha (1), Erica revoluta (1), Erica retorta (1), Erica regia (1), Erica recta (1), Erica pyxidiflora (1), Erica pycnantha (1), Erica psittacine (1), Erica pogonanthera (1), Erica physophylla (1), Erica petricola (1), Erica perlata, Erica pectinifolia (1), Erica patersonii (1), Erica parviporandra (1), Erica pageana (1), Erica oreotragus (1), Erica oblongiflora (1), Erica nana (1), Erica nabea (2), Erica myriadenia (1), Erica multumbellifera (1), Erica monsoniana (1), Erica monadelphia (1), Erica modesta (1), Erica melastoma (1), Erica margaritacea (1), Erica malmesburiensis (1), Erica madida (1), Erica leucotrachela (1), Erica leucopelta (1), Erica leptopus (1), Erica lambertii (1), Erica lachnaeifolia (1), Erica kingaensis (1), Erica karwyderi (1), Erica junonia (1), Erica jasminiflora (1), Erica ingeana (1), Erica infundibuliformis (1), Erica inflata (2), Erica incarnata (1), Erica humifusa (1), Erica holosericea (1), Erica hibbertii (1), Erica hendricksei (1), Erica heleogena (1), Erica goudotiana (1), Erica glomiflora (1), Erica gibbosa (2), Erica georgica (1), Erica garciae (1), Erica frigida (1), Erica filipendula (1), Erica fausta (1), Erica fairii (1), Erica esteriana (1), Erica esterhuyseniae (1), Erica equisetifolia (2), Erica duthieae (1), Erica drakensbergensis (1), Erica densifolia (1), Erica cyathiformis (1), Erica curviflora (1), Erica cristata (1), Erica corifolia (2), Erica condensata (1), Erica coarctata (2), Erica clavisepala (1), Erica chrysocodon (1), Erica chionodes (1), Erica cetrata (1), Erica capitata (1), Erica canescens (1), Erica cameronii (1), Erica caffrorum (1), Erica caffra (1), Erica caespitosa (1), Erica bruniifolia (1), Erica bolusiae (1), Erica blenna (2), Erica bicolor (1), Erica benguelensis (1), Erica baueri (1), Erica banksii (2), Erica axillaris (1), Erica atherstonei (1), Erica annectens (1), Erica albertyniae (1), Erica albens (1), Erica abietina (2), Erica x stuartii (1), Erica vagans (3), Erica umbellata (3), Erica trimera (1), Erica tetralix (6), Erica terminalis (2), Erica sicula (9), Erica scoparia (3), Erica platycodon (4), Erica oatesii (1), Erica multiflora (3), Erica manipuliflora (3), Erica mackaiana (5), Erica lusitanica (3), Erica halicacaba (1), Erica ciliaris (6), Erica carnea (4), Erica bojeri (1), Erica azorica (2), Erica arborea (12), Erica andevalensis (1), Erica scytophylla (1), Erica plukenetii (10). |
| 0 ; 2 | No. species: 2  Family: Ericaceae  (*Erica* = 2). | Erica kingaensis (1), Erica whyteana (1), |
| 1 ; 1 | No. species: 7  Family: Ericaceae  (*Erica* = 7). | Erica sagittata (1), Erica ciliaris (1), Erica urna-viridis (1), Erica physodes (1), Erica erigena (4), Erica australis (4), Erica arborea (1), |
| 2 ; 0 | No. species: 1  Family: Ericaceae  (*Erica* = 1). | Erica maderensis (1), |
| 1 ; 2 | No. species: 2  Family: Ericaceae  (*Erica* = 2). | Erica caffrorum (1), Erica muscosa (1), |
| 3 ; 1 | No. species: 1  Family: Cercidiphyllaceae (*n* = 1). | Cercidiphyllum japonicum (1) |
| 2 ; 2 | No. species: 1  Family Ericaceae  (*Erica* = 1). | Erica agglutinans (1), |
| 2 ; 3 | No. species: 1  Family: Primulaceae (*n* = 1). | Lysimachia thyrsiflora (1), |
| 3 ; 2 | No. species: 126  Family: Hamamelidaceae (*n* = 72),  Sabiaceae (*n* = 32),  Daphniphyllaceae (*n* = 10),  Francoaceae (*n* = 3),  Rubiaceae (*n* = 3),  Pentaphylacaceae (*n* = 2),  Cercidiphyllaceae (*n* = 2),  Menispermaceae (*n* = 1),  Ebenaceae (*n* = 1). | Loropetalum chinense (15), Corylopsis pauciflora (9), Corylopsis stenopetala (1), Corylopsis yunnanensis (2), Corylopsis glabrescens (4), Corylopsis sinensis (5), Sycopsis sinensis (5), Fortunearia sinensis (4), Daphniphyllum macropodum (7), Cercidiphyllum japonicum (10), Mytilaria laosensis (4), Albertisia porcata (2), Cercidiphyllum magnificum (1), Disanthus cercidifolius (7), Rhodoleia parvipetala (2), Distylium myricoides (5), Distylium chinense (2), Distylium macrophyllum (2), Rhodoleia championii (5), Exbucklandia tonkinensis (15), Exbucklandia populnea (14), Exbucklandia longipetala (3), Exbucklandia sp. (5), Daphniphyllum calycinum (4), Daphniphyllum oldhamii (3), Distyliopsis tutcheri (2), Distyliopsis dunnii (4), Corylopsis multiflora (4), Corylopsis glandulifera (2), Corylopsis spicata (2), Distylium tsiangii (1), Parrotia persica (3), Parrotia subaequalis (4), Fothergilla gardenii (2), Hamamelis x intermedia (1), Noahdendron nicholasii (2), Ostrearia australiana (2), Molinadendron sinaloense (1), Sinowilsonia henryi (5), Eustigma oblongifolium (4), Maingaya malayana (2), Matudaea trinervia (1), Chunia bucklandioides (4), Rhodoleia henryi (3), Corylopsis coreana (1), Diospyros montana (1), Hamamelis mollis (5), Sycopsis triplinervia (1), Daphniphyllum laurinum (1), Daphniphyllum glaucescens (9), Daphniphyllum dichotomum (1), Daphniphyllum buchananiifolium (2), Daphniphyllum borneense (1), Corylopsis gotoana, Hamamelis japonica (4), Corylopsis veitchiana (1), Meliosma myriantha (1), Eustigma balansae (1), Meliosma aff. cuneifolia (1), Fothergilla sp. (3), Parrotiopsis jacquemontiana (3), Hamamelis sp. (1), Fothergilla latifolia (1), Ophiocaryon maguirei (1), Ophiocaryon klugii (1), Ophiocaryon heterophyllum (1), Meliosma veitchiorum (1), Meliosma subcordata (1), Meliosma rigida (1), Meliosma rhoifolia (1), Meliosma pinnata (1), Meliosma oldhamii (1), Meliosma occidentalis (1), Meliosma longepedicellata (1), Meliosma irazuensis (1), Meliosma idiopoda (1), Meliosma herbertii (1), Meliosma gracilis (1), Meliosma glandulosa (1), Meliosma glabrata (1), Meliosma flexuosa (1), Meliosma donnellsmithii (1), Meliosma dilleniifolia (1), Meliosma dentata (1), Meliosma cuneifolia (1), Meliosma cornejoi (1), Meliosma clandestina (1), Meliosma callicarpifolia (1), Meliosma caballeroensis (1), Meliosma boliviensis (1), Meliosma angustifolia (1), Meliosma allenii (1), Meliosma alba (4), Corylopsis stelligera (1), Corylopsis trabeculosa (1), Daphniphyllum longeracemosum (1), Distylium dunnianum (2), Distylium pingpienense (2), Distyliopsis laurifolia (1), Corylopsis willmottiae (1), Distylium buxifolium (3), Corylopsis platypetala (1), Distylium racemosum (4), Corylopsis glaucescens (1), Daphniphyllum chartaceum (1), Corylopsis microcarpa (1), Corylopsis velutina (1), Plocama crocyllis (3), Plocama tinctoria (1), Distylium lepidotum (3), Distylium elaeagnoides (1), Distylium cuspidatum (1), Distylium gracile (1), Balbisia meyeniana (1), Balbisia peduncularis (1), Symplococarpon purpusii (1), Symplococarpon hintonii (1), Hamamelis mexicana (1), Dicoryphe stipulacea (2), Gaillonia crocyllis (1), Melianthus gariepinus (1), Hamamelis gariepinus (1), Shaniodendron subaequale (1), Neostrearia fleckeri (1), Molinadendron guatemalense (1), Fothergilla major (1), |
| 4 ; 2 | No. species: 73  Family: Rubiaceae (*n* = 63), Hamamelidaceae (*n* = 3), Geraniaceae (*n* = 3), Sabiaceae (*n* = 2), Dilleniaceae (*n* = 1). | Coptosapelta flavescens (3), Plocama pendula (12), Hedyotis ovata (2), Neostrearia fleckeri (1), Dillenia excelsa (1), Sericanthe andongensis (5), Houstonia palmeri (2), Houstonia acerosa (1), Hekistocarpa minutiflora (4), Timonius mollis (1), Timonius korrensis (1), Timonius salsedoi (1), Timonius subauritus (1), Hedyotis puberulifolia (1), Hedyotis nankunshanensis (1), Petitiocodon parviflorum (3), Hedyotis sp. (8), Hedyotis matthewii (1), Hedyotis nanlingensis (1), Hedyotis longiexserta (1), Hedyotis prostrata (1), Hedyotis interrupta (1), Hedyotis exserta (1), Hedyotis baotingensis (1), Tocoyena arenicola (1), Hedyotis hainanensis (1), Neanotis wightiana (1), Neanotis kwangtungensis (1), Hedyotis yunnanensis (1), Hedyotis yangchunensis (2), Hedyotis xanthochroa (2), Hedyotis wuzhishanensis (2), Hedyotis uncinella (7), Oldenlandia tenuipes (1), Hedyotis shiuyingiae (4), Hedyotis shenzhenensis (1), Hedyotis pulcherrima (1), Hedyotis platystipula (1), Hedyotis paridifolia (1), Hedyotis macrostegia (2), Hedyotis longipetala (1), Hedyotis effusa (1), Hedyotis cryptantha (1), Oldenlandia consanguinea (1), Hedyotis communis (1), Hedyotis caudatifolia (2), Hedyotis cantoniensis (2), Oldenlandia bodinieri (3), Oldenlandia assimilis (1), Hedyotis acutangular (2), Hedyotis vachellii (4), Hedyotis loganioides (2), Hedyotis consanguinea isolate (9), Hedyotis bodinieri (4), Hedyotis bracteosa (2), Hedyotis assimilis (2), Sericanthe odoratissima (2), Psilanthus lebrunianus (1), Rhodoleia championii (1), Ixora peruviana (1), Meliosma veitchiorum (1), Valantia hispida (1), Coptosapelta tomentosa (2), Coptosapelta diffusa (2), Coptosapelta montana (1), Paederia pospischilii (1), Meliosma cuneifolia (1), Pelargonium vinaceum (1), Pelargonium petroselinifolium (1), Pelargonium moniliforme (1), Sinoadina racemosa (1), Matudaea trinervia (1), |
| 3 ; 3 | No. species: 63  Family:  Solanaceae (*n* = 31), Rubiaceae (*n* = 26), Daphniphyllaceae (*n* = 2),  Primulaceae (*n* = 2), Hamamelidaceae (*n* = 1), Olacaceae (*n* = 1), | Lysimachia maritima (1), Plocama botschantzevii (2), Physalis minima (1), Physalis pubescens (3), Physalis angulata (3), Physalis chenopodifolia (1), Withania adpressa (1), Physalis walteri (1), Physalis viscosa (2), Physalis virginiana (1), Physalis victoriana (1), Physalis purpurea (1), Physalis pumila (1), Physalis pruinosa (2), Physalis patula (1), Physalis orizabae (1), Physalis mollis (1), Physalis longifolia (2), Physalis lagascae (1), Physalis ixocarpa (2), Physalis fendleri (1), Physalis x elliottii (1), Physalis cordata (2), Physalis cinerascens (3), Physalis chenopodiifolia (1), Physalis angustifolia (1), Ardisia palmana (1), Physalis peruviana (4), Aptandra tubicina (1), Physalis ampla (1), Daphniphyllum dichotomum (1), Daphniphyllum borneense (1), Physalis ixocarpa (1), Wendlandia glabrata (1), Physalis philadelphica (4), Oldenlandia boscii (2), Ixora sp. (1), Plocama calycoptera (2), Plocama puberula (2), Plocama olivieri (1), Plocama eriantha (2), Plocama crucianelloides (1), Plocama bruguieri (4), Plocama afghanica (6), Plocama somaliensis (1), Plocama hymenostephana (1), Plocama aucheri (2), Plocama macrantha (2), Plocama dubia (1), Plocama thymoides (1), Distylium lepidotum (1), Oldenlandia affinis (1), Wendlandia paniculata (1), Macrocnemum roseum (1), Sabicea villosa (1), Physalis heterophylla (1), Margaranthus solanaceus (1), Pseudogaillonia hymenostephana (1), Gaillonia olivieri (1), Gaillonia eriantha (1), Choulettia reboudiana (1), Aitchisonia rosea (1), Jaubertia aucheri (1). |

**Primer-BLAST results for *Erica cinerea* marker with multiple isolates removed. Top 10 results shown, full analysis available on CORD (doi).**

>[KP737419.1](https://www.ncbi.nlm.nih.gov/entrez/viewer.fcgi?db=nucleotide&id=815932660) Erica cinerea tRNA-Leu (trnL) gene, partial sequence; trnL-trnF intergenic spacer, complete sequence; and tRNA-Phe gene, partial sequence; chloroplast

product length = 101

Forward primer 1 CTCCATTGTCTACTAGATCTTTTGA 25

Template 373 ......................... 397

Reverse primer 1 CAATAAATTTCATTGTTGTCGTCA 24

Template 473 ........................ 450

>[PV443244.1](https://www.ncbi.nlm.nih.gov/entrez/viewer.fcgi?db=nucleotide&id=2954826118) Erica mannii subsp. usambarensis voucher Bytebier, B. s.n. (NBG) tRNA-Leu (trnL) gene, partial sequence; chloroplast

product length = 101

Forward primer 1 CTCCATTGTCTACTAGATCTTTTGA 25

Template 368 ......................... 392

Reverse primer 1 CAATAAATTTCATTGTTGTCGTCA 24

Template 468 .....................G.. 445

>[KY110838.1](https://www.ncbi.nlm.nih.gov/entrez/viewer.fcgi?db=nucleotide&id=1241824563) Erica viscaria subsp. longifolia voucher NBG:Hitchcock A 91 /04 tRNA-Leu (trnL) gene, partial sequence; trnL-trnF intergenic spacer and tRNA-Phe gene, complete sequence; and trnF-ndhJ intergenic spacer, partial sequence; plastid

product length = 101

Forward primer 1 CTCCATTGTCTACTAGATCTTTTGA 25

Template 367 ......................... 391

Reverse primer 1 CAATAAATTTCATTGTTGTCGTCA 24

Template 467 .....................G.. 444

>[KY110832.1](https://www.ncbi.nlm.nih.gov/entrez/viewer.fcgi?db=nucleotide&id=1241824554) Erica stokoei voucher NBG:62/83 tRNA-Leu (trnL) gene, partial sequence; trnL-trnF intergenic spacer, tRNA-Phe gene, and trnF-ndhJ intergenic spacer, complete sequence; and NADH-plastoquinone oxidoreductase subunit J (ndhJ) gene, partial cds; plastid

product length = 101

Forward primer 1 CTCCATTGTCTACTAGATCTTTTGA 25

Template 371 ......................... 395

Reverse primer 1 CAATAAATTTCATTGTTGTCGTCA 24

Template 471 .....................G.. 448

>[KY110831.1](https://www.ncbi.nlm.nih.gov/entrez/viewer.fcgi?db=nucleotide&id=1241824553) Erica phaeocarpa voucher NBG:SANBI 543 /87 tRNA-Leu (trnL) gene, partial sequence; trnL-trnF intergenic spacer, complete sequence; and tRNA-Phe gene, partial sequence; plastid

product length = 101

Forward primer 1 CTCCATTGTCTACTAGATCTTTTGA 25

Template 373 ......................... 397

Reverse primer 1 CAATAAATTTCATTGTTGTCGTCA 24

Template 473 .....................G.. 450

>[KY110828.1](https://www.ncbi.nlm.nih.gov/entrez/viewer.fcgi?db=nucleotide&id=1241824548) Erica parilis voucher NBG:Hitchcock A 97 /04 tRNA-Leu (trnL) gene, partial sequence; trnL-trnF intergenic spacer, complete sequence; and tRNA-Phe gene, partial sequence; plastid

product length = 101

Forward primer 1 CTCCATTGTCTACTAGATCTTTTGA 25

Template 371 ......................... 395

Reverse primer 1 CAATAAATTTCATTGTTGTCGTCA 24

Template 471 .....................G.. 448

>[KY110827.1](https://www.ncbi.nlm.nih.gov/entrez/viewer.fcgi?db=nucleotide&id=1241824546) Erica nevillei voucher NBG:Hitchcock A 86 /04 tRNA-Leu (trnL) gene, partial sequence; trnL-trnF intergenic spacer, tRNA-Phe gene, and trnF-ndhJ intergenic spacer, complete sequence; and NADH-plastoquinone oxidoreductase subunit J (ndhJ) gene, partial cds; plastid

product length = 101

Forward primer 1 CTCCATTGTCTACTAGATCTTTTGA 25

Template 371 ......................... 395

Reverse primer 1 CAATAAATTTCATTGTTGTCGTCA 24

Template 471 .....................G.. 448

>[KY110823.1](https://www.ncbi.nlm.nih.gov/entrez/viewer.fcgi?db=nucleotide&id=1241824541) Erica situshiemalis voucher NBG:Oliver EGH s.n. tRNA-Leu (trnL) gene, partial sequence; trnL-trnF intergenic spacer and tRNA-Phe gene, complete sequence; and trnF-ndhJ intergenic spacer, partial sequence; plastid

product length = 101

Forward primer 1 CTCCATTGTCTACTAGATCTTTTGA 25

Template 371 ......................... 395

Reverse primer 1 CAATAAATTTCATTGTTGTCGTCA 24

Template 471 .....................G.. 448

>[OP172987.1](https://www.ncbi.nlm.nih.gov/entrez/viewer.fcgi?db=nucleotide&id=2501426976) Erica walkeria voucher ? 140 (NBG) tRNA-Leu (trnL) gene, trnL-trnF intergenic spacer, tRNA-Phe (trnF) gene, and trnF-ndhJ intergenic spacer, complete sequence; and ndhJ gene, partial sequence; chloroplast

product length = 101

Forward primer 1 CTCCATTGTCTACTAGATCTTTTGA 25

Template 371 ......................... 395

Reverse primer 1 CAATAAATTTCATTGTTGTCGTCA 24

Template 471 .....................G.. 448

>[OP172983.1](https://www.ncbi.nlm.nih.gov/entrez/viewer.fcgi?db=nucleotide&id=2501426972) Erica venustiflora subsp. glandulosa voucher Pirie, M.D. 1284 (NBG) tRNA-Leu (trnL) gene, complete sequence; trnL-trnF intergenic spacer and tRNA-Phe (trnF) gene, partial sequence; trnF-ndhJ intergenic spacer, complete sequence; and ndhJ gene, partial sequence; chloroplast

product length = 101

Forward primer 1 CTCCATTGTCTACTAGATCTTTTGA 25

Template 365 ......................... 389

Reverse primer 1 CAATAAATTTCATTGTTGTCGTCA 24

Template 465 .....................G.. 442
